# Natural variations in MdBPM2/MdRGLG3‐MdNAC83 network controlling the quantitative segregation of apple fruit storability

**DOI:** 10.1111/jipb.70044

**Published:** 2025-10-01

**Authors:** Bei Wu, Fei Shen, Ziying Zhou, Wenhui Ren, Yi Wang, Ting Wu, Zhenhai Han, Xinzhong Zhang

**Affiliations:** ^1^ College of Horticulture China Agricultural University Beijing 100193 China

**Keywords:** fruit storability, genetic variation, *Malus domestica* Borkh, protein ubiquitination, quantitative trait

## Abstract

Dissecting quantitative traits into Mendelian factors is a great challenge in genetics. Apple fruit storability is a complex trait controlled by multi‐genes with unequal effects. We previously identified 62 quantitative trait loci (QTLs) associated with apple fruit storability and genomics‐assisted prediction (GAP) models were trained using 56 QTL‐based markers. Here, three candidate genes, *MdNAC83*, *MdBPM2*, and *MdRGLG3*, were screened from the regions of QTLs with large *G*’ value and large genetic effects. Both a 216‐bp deletion and an SNP934 T/C at the promoter of *MdNAC83* were associated with higher *MdNAC83* expression but an SNP388 G/A at the coding region significantly reduced the activity to activate the expression of the target genes *MdACO1*, *MdMANA3*, and *MdXTH28*. MdBPM2 and MdRGLG3 participated in the ubiquitination of MdNAC83. SNP657 T/A of *MdBPM2* and SNP167 C/G of *MdRGLG3* caused a reduction in the activity to ubiquitinate MdNAC83. By the addition of functional markers to the GenoBaits SNP array, the prediction accuracy of the updated GAP models increased to 0.7723/0.6231 and 0.5639/0.5345 for flesh firmness/crispness at harvest and flesh firmness/crispness retainability, respectively. The variation network involving eight simple Mendelian variations in six genes helps to gain insight into the molecular quantitative genetics, to improve breeding strategy, and to provide targets for future genome editing.

## Graphical abstract

During postharvest cold storage of apples, the NAC transcription factor MdNAC83 binds to the promoters and activates the expression of downstream genes and is regulated by E3 ligases, which promote its ubiquitination and degradation. Genetic variants in *MdNAC83* and its downstream genes form a network to regulate apple fruit storability.

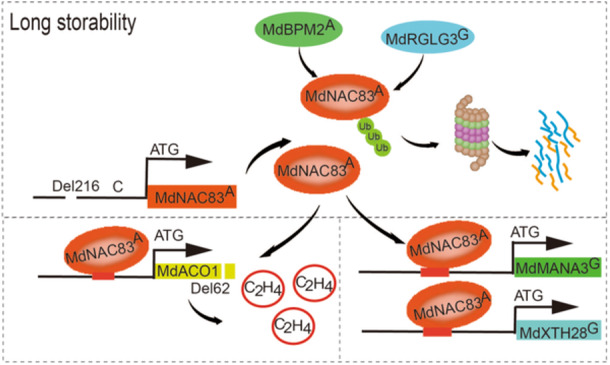

## INTRODUCTION

In both plants and animals, many economically important traits are quantitatively inherited, the quantitative traits are usually affected by a group of genetic variations with minor effect and with complicated allelic or nonallelic interactions (Li et al., 2018; Sun et al., 2022; Fu et al., 2023). Dissection of a quantitative trait locus (QTL) into a certain number of Mendelian factors has long been a great challenge for genetics.

Apple (*Malus domestica* Borkh.) is a representative climatic fruit and apple fruit cold storability is a typical quantitative trait on which many efforts have been made over decades to understand the genetic network. QTLs for apple fruit storability were repeatedly reported on all the chromosomes except chr04, chr07, chr09, and chr13 (King et al., 2001; Longhi et al., 2012; Bink et al., 2014; Wu et al., 2021a; 2021b). It was established that the gaseous phytohormone ethylene is the key player to regulate flesh softening during cold storage (Alexander and Grierson, 2002; Grierson, 2013). Large numbers of functional variations affecting apple flesh softening have been identified and experimentally validated, including variations in the genes involved in ethylene synthesis, such as *MdACO* and *MdACS* (Sunako et al., 1999; Harada et al., 2000; Costa et al., 2005; Oraguzie et al., 2004; 2007; Sharma et al., 2021), ethylene signaling, e.g., *MdERF*s (Li et al., 2016; Hu et al., 2020; Wu et al., 2021b), and the downstream cell wall metabolism, such as *MdPG1*, *MdPME1*, *MdPAE10*, and *MdExp7* (Amyotte et al., 2017; Wu et al., 2021a). Instead of ethylene, cross‐talk between abscisic acid (ABA) and ethylene has also been reported to participate in the regulation of fruit firmness and storability. A genome‐wide association study (GWAS) of *Malus* accessions revealed that a single nucleotide polymorphism (SNP) in *MdNAC18.1*, an ABA pathway gene, was significantly related to apple fruit firmness (Larsen et al., 2019). *SlNAC1* and *SlNAC4* have been reported to negatively regulate ethylene synthesis and fruit firmness in tomato (*Solanum lycopersicum* L.) (Klee and Giovannoni, 2011; Ma et al., 2014; Zhu et al., 2014; Meng et al., 2016; Kumar et al., 2018). The NAC transcription factor functions by binding to the promoter of key fruit ripening and flesh softening genes such as *SlACS2*, *SlACS4*, *SlPG2a*, *SlXET*, and *SlEXP1*, thereby positively or negatively regulating ethylene synthesis and cell wall metabolism (Lü et al., 2018; Shinozaki et al., 2018; Gao et al., 2018; 2020; Liu et al., 2021).

The complexity of a quantitative trait lies not only in the genetic variation network by transcriptional regulation but sometimes by nonallelic interactions at post‐translational and/or epigenetic levels. Apple skin red coloration represents an ideal example of multilevel regulation. The presence or absence of apple skin red pigmentation is determined by a transposable element (TE) insertion upstream of *MdMYB1* (Zhang et al., 2019). Apple skin color patterns are controlled by epigenetic modulation, variation in the degree of DNA hypermethylation in the *MdMYB10* promoter region affects apple skin color patterns, either in blushing or in striped phenotypes (Telias et al., 2011, Liu et al., 2022a). Nonallelic interaction of genetic variations can often occur at the post‐transcriptional level. The variation A to T SNP at the *MdPP2CH* coding sequences (CDS) affects the ability of dephosphorylation over the target protein MdALMTII (Jia et al., 2018). In apple, the ubiquitination‐related scaffold protein MdBT2 can control malate accumulation and synergistically modulate ethylene biosynthesis and anthocyanin accumulation (Mandadi et al., 2009; An et al., 2020). MdBT2 can also negatively regulate iron homeostasis by interacting with MdNAC1 in apple (Li et al., 2022).

Patterns of allelic interaction may also contribute to the genetic complexity of a quantitative trait. Genetic variations in rate unlimiting genes exert dominant allelic effect causing segregation of only two phenotypes. A 4,097‐bp Gypsy‐like LTR TE insertion −3,297 bp upstream of *MdMYB1* is associated with red‐skinned phenotype in apple, irrespective of homozygous or heterozygous genotypes (Zhang et al., 2019). Similarly, in peach and nectarine (*Prunus persica* L.), trees with a TT genotype of the SNP A/T at *PpCCD4* CDS bear fruit with yellow flesh, the flesh color of those with the AA and AT genotypes is white (Adami et al., 2013; Falchi et al., 2013). Functional variations in rate‐limiting genes usually exhibit an additive or partially dominant allelic effect, resulting in segregation of three phenotypes in the progeny. Significant differences in genotype effects on apple fruit acidity were identified among AA, AT, and TT genotypes of the SNP A/T at *MdPP2CH* CDS (Jia et al., 2018). The partial dominant allelic effect was estimated in most markers for apple fruit weight, ripening date, and soluble solid content (Shen et al., 2022). Interestingly, the allelic effect often varied with cross populations, for example, the SNP G/A at *MdALMTII* CDS showed complete dominant allelic effect in the progeny of ‘Jonathan’ × ‘Golden Delicious’, the genotype effect of GA was the same as that of AA, however, the genotype effect of GA was near the intermediate between GG and AA in the progeny of ‘Zisai Pearl’ × ‘Red Fuji’ (Jia et al., 2018).

For complex quantitative traits, genomic selection (GS) is a powerful breeding tool for both livestock and plants (Meuwissen et al., 2001; Crossa et al., 2017). Genomics‐assisted prediction (GAP) was recently developed by a principle like GS, but GAP used QTL‐based markers instead of genome‐wide high‐density markers (Zheng et al., 2020a; 2020b; Wu et al., 2021b; Shen et al., 2022). Apple fruit storability was previously quantitatively characterized by flesh firmness retainability (FFR) and flesh crispness retainability (FCR), which were quantified by the maximum time (in months) of flesh firmness (>7.0 kg/cm^2^) and flesh crispness (> 0.7 kg/cm^2^) retention during cold storage, respectively (Wu et al., 2021b). Sixty‐two confident QTLs for FFR and FCR were identified using an interspecific hybrid population of *Malus* (Wu et al., 2021b). Within the 62 QTL regions, we previously developed 56 markers and GAP models were trained for apple flesh firmness at harvest (FF), flesh crispness at harvest (FC), FFR, and FCR, the prediction accuracy was relatively higher than that of pure GS with a high‐density SNP array (McClure et al., 2018; Wu et al., 2021b).

Although the GAP models for apple FF, FC, FFR, and FCR have been practically used in apple breeding, the prediction accuracy could still be improved. Addition of functional markers into GS or GAP models led to a significant increase in the predictability (Liabeuf et al., 2018; Ma et al., 2019; Yang et al., 2022; Wen et al., 2024; Zhang et al., 2025). The aims of the present study were to explore functional variations from QTL regions for apple FFR and FCR, in which a complex quantitative trait like apple fruit cold storability could be dissected into simple Mendelian factors. Several functional variations were identified and validated in *MdNAC83*, *MdBPM2*, and *MdRGLG3*. These variations, as well as their allelic and nonallelic interactions, formed a complicated regulatory network contributing to apple FFR and FCR.

## RESULTS

### Screening for candidate genes and allelic variations within QTL regions

**Figure 1:**
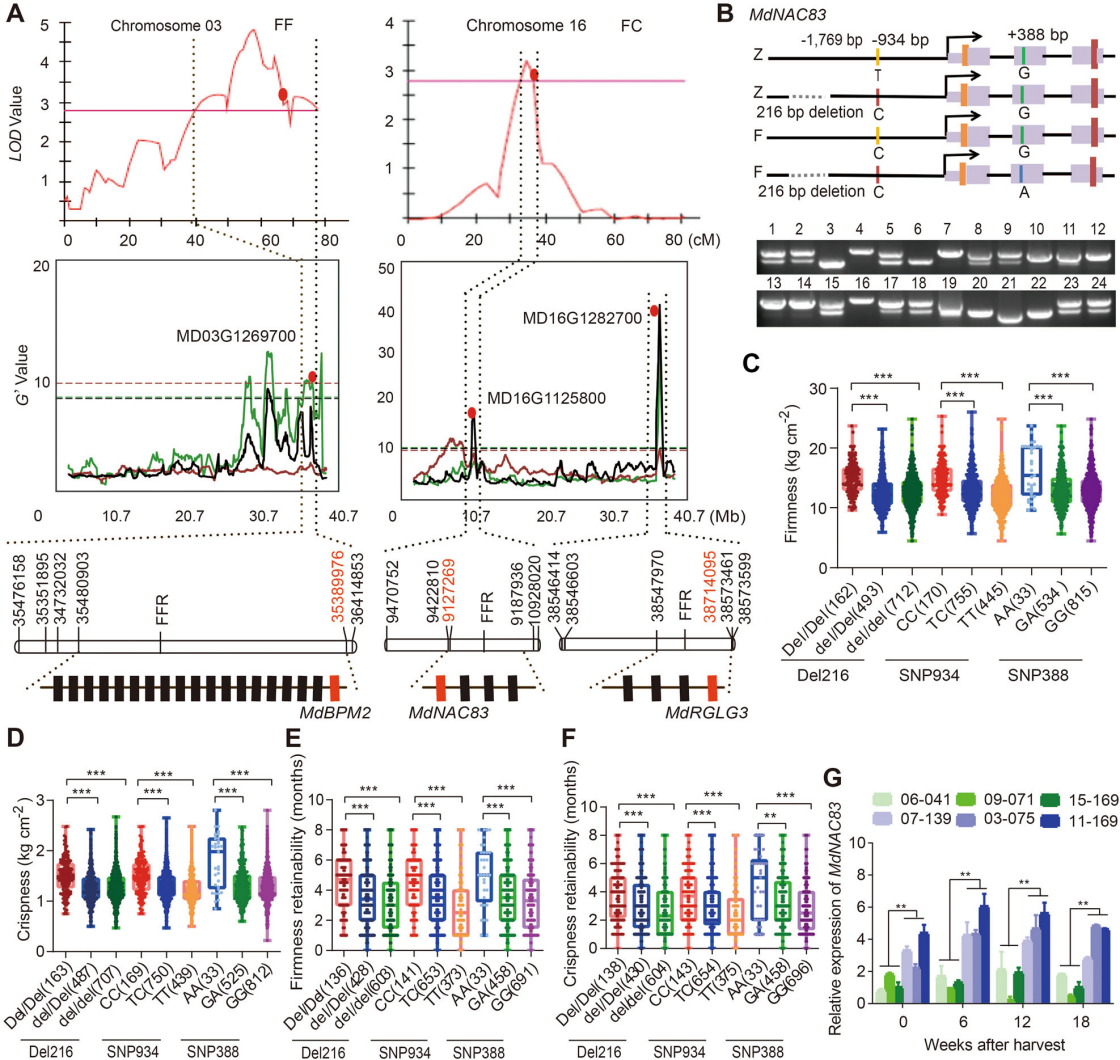
Screening for candidate genes and allelic variations within QTL regions **(A)** QTL identification and narrowing down the QTL intervals using a biparental cross population (*Malus asiatica* Nakai ‘Zisai Pearl’ × *M. domestica* Borkh. ‘Red Fuji’). The upper images show QTLs for apple flesh firmness at harvest (FF) (upper left) on chromosome 3 and for flesh crispness at harvest (FC) (upper right) on chromosome 16 via MapQTL 6.0. The middle images indicate QTLs for flesh firmness retainability (FFR) on chromosome 3 and 16 (middle left and middle right), respectively, by BSATOS software, the dashed lines indicate the parentage of the QTLs, which were mapped on the maternal (red), paternal (green), and both (black) parents. The bottom images show the narrowed‐down intervals of QTLs F03.4, H16.1, and H16.2, respectively, by additional Genobaits markers. The location of the candidate genes at the QTL regions were marked with red filled dots. **(B)** Diagrams and PCR genotyping images showing the genetic variations in *MdNAC83* in ‘Zisai Pearl’ (Z) and ‘Red Fuji’ (F). The 216‐bp deletion of *MdNAC83* was genotyped by PCR in 12 *Malus* accessions (1–12) and 12 full‐sib hybrid lines (13–24). **(C**–**F)** Genotype effects of *MdNAC83* Del216, SNP934 T/C, and SNP388 G/A on flesh firmness at harvest **(C)**, flesh crispness at harvest **(D)**, flesh firmness retainability **(E)**, and flesh crispness retainability **(F)**. **(G)** The expression of *MdNAC83* in hybrid lines with different Del216 genotypes (Del216:Del216 or del216:del216) during cold storage. The Del216 genotype of hybrid lines 03‐075, 07‐139, and 11‐169 was Del216:Del216, while that of 06‐041, 09‐071, and 15‐169 was del216:del216.

Map‐based QTL mapping (by MapQTL 6.0) identified three confident QTLs, F03, F10, and F16, for apple FF or FC were mapped on chromosomes 3, 10, and 16, respectively using the F1 hybrid population derived from ‘Zisai Pearl’ × ‘Red Fuji’ (Figure 1A; Table S1). Of these three QTLs, F03 for FF overlapped with the previously identified QTLs for FFR (via BSA‐seq), F03.1, F03.2, F03.3, and F03.4 for FF whereas F16 for FC covered the interval of the QTL H16.1 for FFR by the previous BSA‐seq (Figure 1A) (Wu et al., 2021b).

The *G’* values of QTLs F03.1, F03.2, F03.4, and H16.1 were considerably large (>10) (Figure 1A). Within the QTL F03.1, functional variation on *MdERF3* has been identified and experimentally validated previously (Wu et al., 2021b). In this study, the regions of the QTLs F03.2, F03.4, and H16.1 were chosen for further experiments. Using an additional five, six, and five GenoBaits markers, respectively, the intervals of QTLs F03.2, F03.4, and H16.1 were narrowed down to 490.5 kb (Chr03_28907974 to Chr03_29398499), 90.9 kb (Chr03_35389976 to Chr03_35480903), and 60.7 kb (Chr16_9127269 to Chr16_9187936) (Figure 1A; Table S2).

In addition to the overlapping QTLs F03.2, F03.4, and H16.1, the *G’* value of QTL H16.2 was as extremely high as 42.43, in this study, the interval of QTL H16.2 was also narrowed down to 166.1 kb (Chr16_38547970 to Chr16_38714095) using six newly designed GenoBaits markers (Figure 1A; Table S2).

Within the narrowed‐down region of the QTL H16.1, four genes, *MdNAC83* (MD16G1125800), *MdMAB1* (MD16G1126100), and two unknown genes (MD16G1125900 and MD16G1126000), were annotated according to the GDDH13 v1.1 apple genome (Table S3). The expression levels of *MdNAC83* were relatively high throughout the post‐harvest storage and *MdNAC83* was thus selected as a candidate gene (Table S3). The marker Chr03_9127269 was designed exactly on the *MdNAC83* promoter and NAC transcription factors were repeatedly reported to be involved in fruit ripening and firmness in both climacteric and non‐climacteric fruit species (Leng et al., 2014; Kumar et al., 2018; Larsen et al., 2019; Chen et al., 2020; Martin‐Pizarro et al., 2021; Wang et al., 2022).

We previously found that the marker SNP9127629 on *MdNAC83* exerted the highest marker effect value (2.59) on FFR (Wu et al., 2021b). The promoter and CDS of *MdNAC83* in ‘Zisai Pearl' and ‘Red Fuji' were cloned and analyzed here by Sanger sequencing. A 216‐bp deletion (Del216, Chr16_ 9126219‐9126434) at the position of −1,769 bp upstream of the ATG codon of *MdNAC83* was found to be heterozygous in both ‘Red Fuji' and ‘Zisai Pearl', which was linked to the previous marker SNP934 T/C (−934 bp, Chr16_9127269) (Figure 1B; Table S4). A heterozygous nonsynonymous SNP388 G/A (+ 388 bp, Chr16_9128903) was also detected at the functional domain of the CDS of *MdNAC83* in ‘Red Fuji’, which could lead to slight changes in the secondary structure of MdNAC83, the α‐helical content was decreased by peptide structure prediction analysis (Figures 1B, S1B; Table S4). *MdNAC83* Del216 was segregated by a ratio of 2:5:5 for *Del216/Del216*: *Del216/del216*: *del216/del216* in 12 *Malus* accessions and a ratio of 4:5:3 in 12 full‐sib hybrid lines from ‘Zisai Pearl’ × ‘Red Fuji’ (Figure 1B). The term del216 here and after represents the absence of the 216 bp deletion in the *MdNAC83* promoter.

*MdNAC83* SNP934 CC genotype was previously reported to convey a positive genotype effect on apple fruit post‐harvest storability (Wu et al., 2021b). In this study, we found that the genotype effects of *MdNAC83* SNP388 AA were relatively higher than those of Del216:Del216 and SNP934 CC on FF, FC, FFR, and FCR, respectively (Figure 1C–F). The joint effects of genotype combinations of *MdNAC83* Del216 and SNP388 AA are significantly higher than those of *MdNAC83* Del216 and SNP388 GG on FF, FC, FFR, and FCR, respectively (Figure S2A–D). The data indicated that variations in both promoter and CDS of *MdNAC83* contributed to the phenotype segregations whereas SNP388 A allele made a greater contribution than Del216. Forty‐five genes were annotated within the narrowed‐down region of QTL F03.2 (Figure S1A; Table S3). The markers designed closely linked to *MdTBC1* (Chr03_27276582) and *MdCRLK1* (Chr03_29398499) exhibited 1.69 and 1.19 months marker effects on FFR and 1.58 and 0.48 months marker effects on FCR (Table S5). Whether there are functional variations in *MdTBC1* and *MdCRLK1* is to be identified and validated in a future study.

The narrowed‐down interval of the QTL F03.4 contained 19 genes including a *MdBTs* homologous gene *MdBPM2* (MD03G1269700) (Table S3). *MdBTs* have been reported to regulate fruit ripening in apples (An et al., 2020). We previously found that the marker SNP31718792 linked to *MdBPM2* showed a relatively high marker effect value (2.45) on FCR (Wu et al., 2021b). MdBPM2 was predicted to interact with MdNAC83 through bioinformatics analysis using a multi‐dimensional omics database‐AppleMDO (Da et al., 2019).

Four genes, *MdWDR5A* (MD16G1282400), *MdbHLH25* (MD16G1282500), *MdRGLG3* (MD16G1282700), and an unknown gene (MD16G1282600), were annotated based on the GDDH13.1 apple genome within the narrowed‐down interval of the QTL H16.2 (Table S3). The functional variations in *MdWDR5A* and *MdbHLH25* and their effects on FF and FC have been identified and validated previously (Yang et al., 2022). MdRGLG3 is a member of the RGLG E3 ubiquitin ligase superfamily, which was reported to regulate ABA signaling and thus affect fruit ripening and flesh firmness in tomato (Wu et al., 2016; Wang et al., 2021). We previously reported that the marker SNP38573461, linked to *MdRGLG3*, exerted a high marker effect value (2.18) on FFR (Wu et al., 2021b).

No significant differences in the expression of *MdBPM2* and *MdRGLG3* were detected between apples with long and short FFR or FCR during cold storage using RNA‐seq assay (Wu et al., 2021b). However, a nonsynonymous SNP657 T/A (+ 657 bp, Chr03_35391111) at the BT‐POZ domain in the CDS of *MdBPM2* was detected in ‘Zisai Pearl’ in this study (Table S4). *MdBPM2* SNP657 TT genotypes exerted a negative genotype effect on FF, FC, FFR, and FCR (Figure S3). Similarly, a heterozygous nonsynonymous SNP167 C/G (+ 167 bp, Chr16_38704799) was detected at the VWFA superfamily domain in the CDS of *MdRGLG3* in ‘Zisai Pearl’ and ‘Red Fuji’ (Table S4). *MdRGLG3* SNP167 GG genotype exerted a positive effect on FF, FC, FFR, and FCR, whereas a significant negative genotype effect of SNP167 CC was observed (Figure S3).

### Functional validation of allelic variations in *MdNAC83*

**Figure 2:**
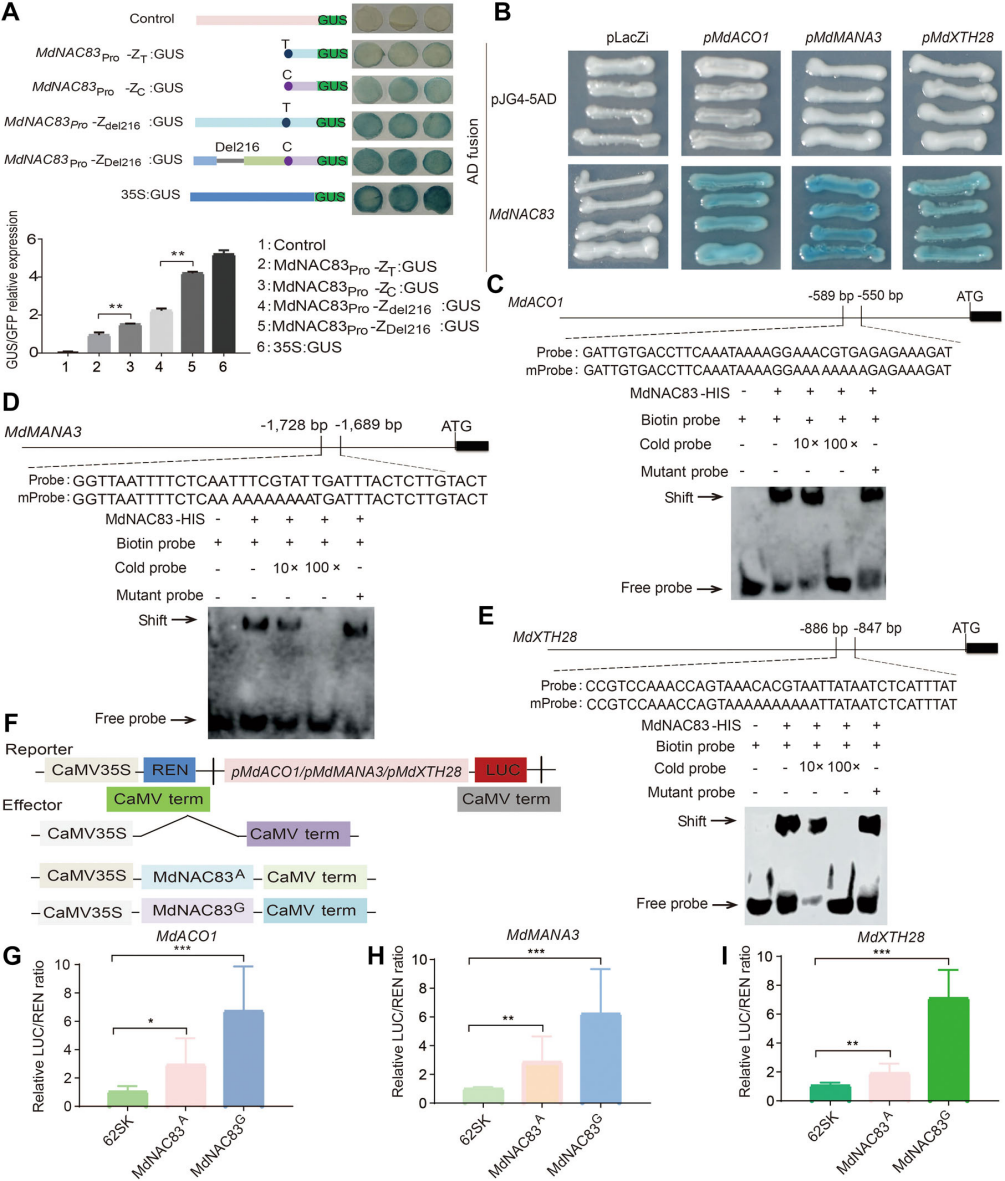
**Impact of natural variations in** ***MdNAC83*** **on the promoter activity or the interaction with the promoters of downstream target genes,** ***MdACO1, MdMANA3***, **and** ***MdXTH28*** **(A)** β‐Glucuronidase (GUS) staining and GUS/GFP relative expression demonstrating the promoter activity *MdNAC83* with or without Del216 and SNP934 C allele. **(B–E)** Yeast‐one‐hybrid (Y1H) **(B)** and electrophoretic mobility shift assay (EMSA) showing that MdNAC83 bound directly to the promoter regions of *MdACO1* **(C)**, *MdMANA3* **(D)**, and *MdXTH28* **(E)**. The upper parts of panels **(C–E)** show the locations and the sequences of MdNAC83 binding sites, “[TA][TG][AGC]CGT[GA][TA]”. Competition for binding was performed with 10× and 100× competitor probes containing the NAC binding sites or mutant sites. The symbol “–” represents absence of competitor probe. **(F–I)** Transient luciferase assays demonstrating that MdNAC83 induced *MdACO1* **(G)**, *MdMANA3* **(H)**, and *MdXTH28* **(I)** expression. Error bars represent ± *SD* of three biological replicates. Statistically significant differences were determined by *t*‐tests: (*P < 0.05, **P < 0.01, ***P < 0.001*)*. Empty vector (pGreen II62‐SK) with each reporter was set as the control and normalized as 1. The relative luciferase/Renilla (LUC/REN) ratio equals the ratio of MdNAC83^A/G^ with each reporter/control.

The previous RNA‐seq data showed that, throughout the 18 weeks of cold storage, the expression of *MdNAC83* was higher in apples of hybrid plants with Del216 homozygous or heterozygous genotypes than with the del216 homozygous genotype (Figure S2E) (Wu et al., 2021b). In the mesocarp of hybrid plants with *MdNAC83* Del216 heterozygous, the number of *MdNAC83* RNA‐seq transcripts with SNP388 A (linked to Del216) was significantly higher than that with SNP388 G (linked to del216) during storage (Figure S2F) (Wu et al., 2021b). In this study, qRT‐PCR assay confirmed that the relative expression of *MdNAC83* in apple mesocarp of hybrid lines (07‐139, 03‐075 and 11‐169) with Del216 homozygous genotype was significantly higher than that (06‐041, 09‐071, and 15‐169) with del216 homozygous genotype during post‐harvest cold storage (Figure 1G). To determine whether the promoter of *MdNAC83* containing Del216 exhibited higher transcription activity, a β‐glucuronidase (GUS) reporter assay was performed in tobacco (*Nicotiana benthamiana*) leaves. GUS/GFP relative expression data in the GUS reporter assay revealed that the promoter activity of the *MdNAC83* Del216 allele was significantly higher than that of the del216 allele, whereas the *MdNAC83* SNP934 T allele exhibited a reduced promoter activity than the SNP934 C allele (Figure 2A). These data confirmed that the expression of *MdNAC83* with Del216 genotype was higher than with del216 genotype.

Using the previous RNA‐seq data between samples with different FFR or FCR (Wu et al., 2021b), a co‐expression network analysis was performed using the genes from the yellow (2,094 genes), magenta (69 genes), and green (91 genes) modules. As a result, 146 genes were found in a co‐expression network (Figure S4B; Table S6). Weighted correlation network analysis (WGCNA) indicated that *MdNAC83* was included in the yellow module. Thirty genes were predicted to be directly regulatory partners of *MdNAC83* according to the AppleMDO database (http://bioinformatics.cau.edu.cn/AppleMDO/index.php). Five of the 30 genes were both differentially expressed genes and within QTL regions, including *MdACO1*(MD10G1328100), *MdXTH28* (MD16G1014000), *MdERF118* (MD16G1043500), *MdMANA3* (MD02G1129000), and *MdNAC83* (Table S6). NAC family transcription factor usually binds to the core motif “[TA][TG][AGC]CGT[GA][TA]” of the promoter of the target genes (O'Malley et al., 2016). Sequence analysis indicated that there was at least one NAC binding motif at the 2.0 kb upstream regions of *MdACO1*, *MdMANA3*, and *MdXTH28*. Subsequent yeast‐one‐hybrid (Y1H) and electrophoretic mobility shift (EMSA) assays confirmed that MdNAC83 directly bound to the promoter of *MdACO1*, *MdbMANA3*, and *MdXTH28*, respectively (Figure 2B–E). *MdMANA3* and *MdXTH28* were located within the QTL region of C‐F2.1 and F‐Z16.1 (Wu et al., 2021b), the allelic variations in *MdACO1*, *MdMANA3*, and *MdXTH28* were confirmed by Sanger sequencing (Table S4). Luciferase assay (LUC) indicated that the *MdNAC83* SNP388 A allele displayed significantly reduced effects compared with the SNP388 G allele on inducing the expression of *MdACO1*, *MdMANA3*, and *MdXTH28* (Figure 2F–I).

**Figure 3:**
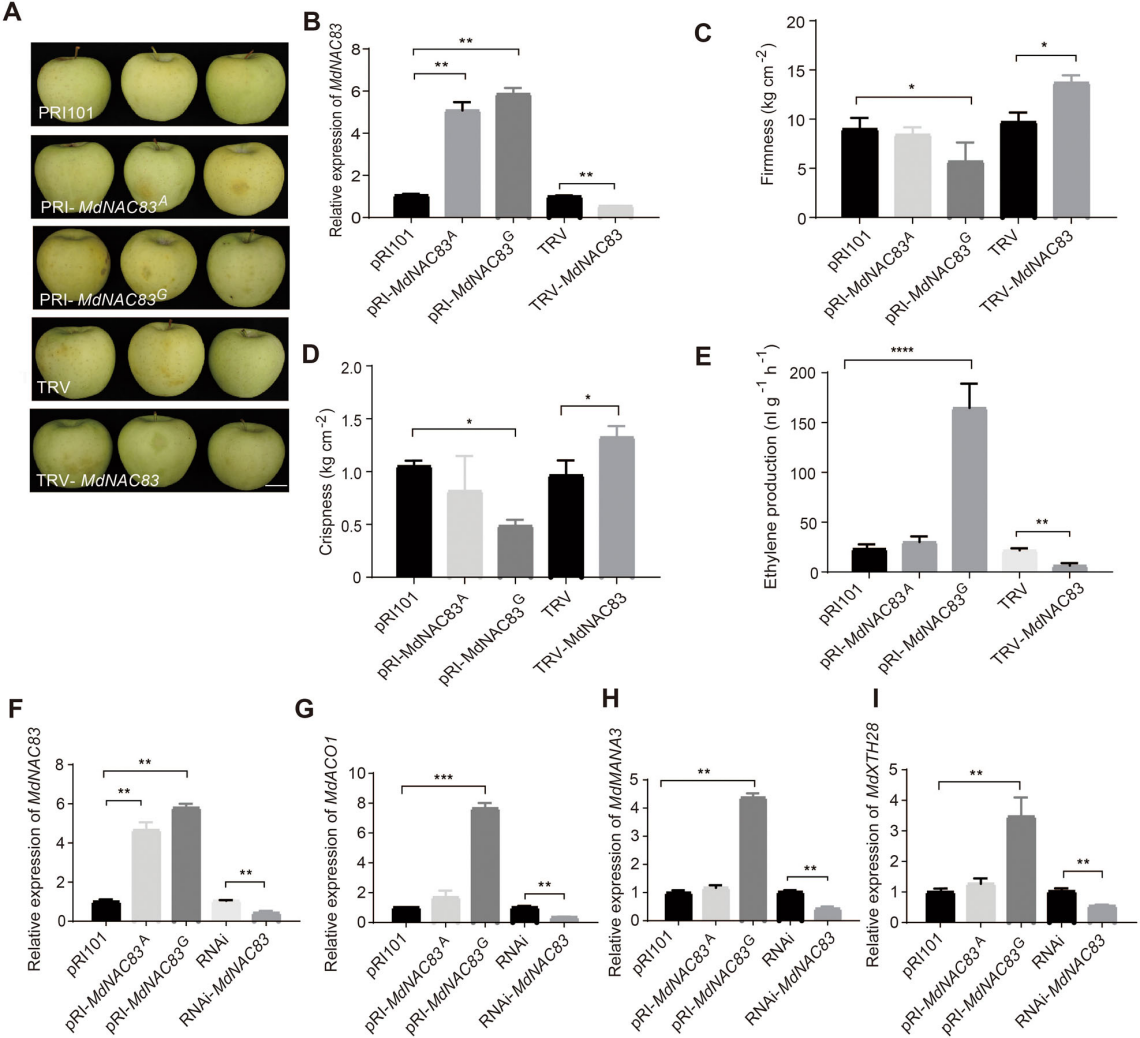
**Transient over‐expression or virus‐induced gene silencing of** ***MdNAC83*** **in “Golden Delicious” and transgenic apple calli** **(A)** Photographs showing changes in appearance phenotype after transient transformations in ‘Golden Delicious’. Scale bar, 3 mm. **(B)** The expression of *MdNAC83* over‐expression (*pRIMdNAC83*) or silencing (*TRVMdNAC83*) in ‘Golden Delicious’. **(C**–**E)** Changes in flesh firmness **(C)**, flesh crispness **(D)**, and ethylene production rate **(E)** in transiently transformed apples. **(F**–**I)** The expression of *MdNAC83* **(F)**, *MdACO1* **(G)**, *MdMANA3* **(H)**, and *MdXTH28* **(I)** in transgenic apple calli over‐expressing *pRIMdNAC83* or *MdNAC83*‐RNAi lines. PRI101 and RNAi as untransformed control. Asterisks indicate statistical significance (*P < 0.05, **P < 0.01, ***P < 0.001).

**Figure 4:**
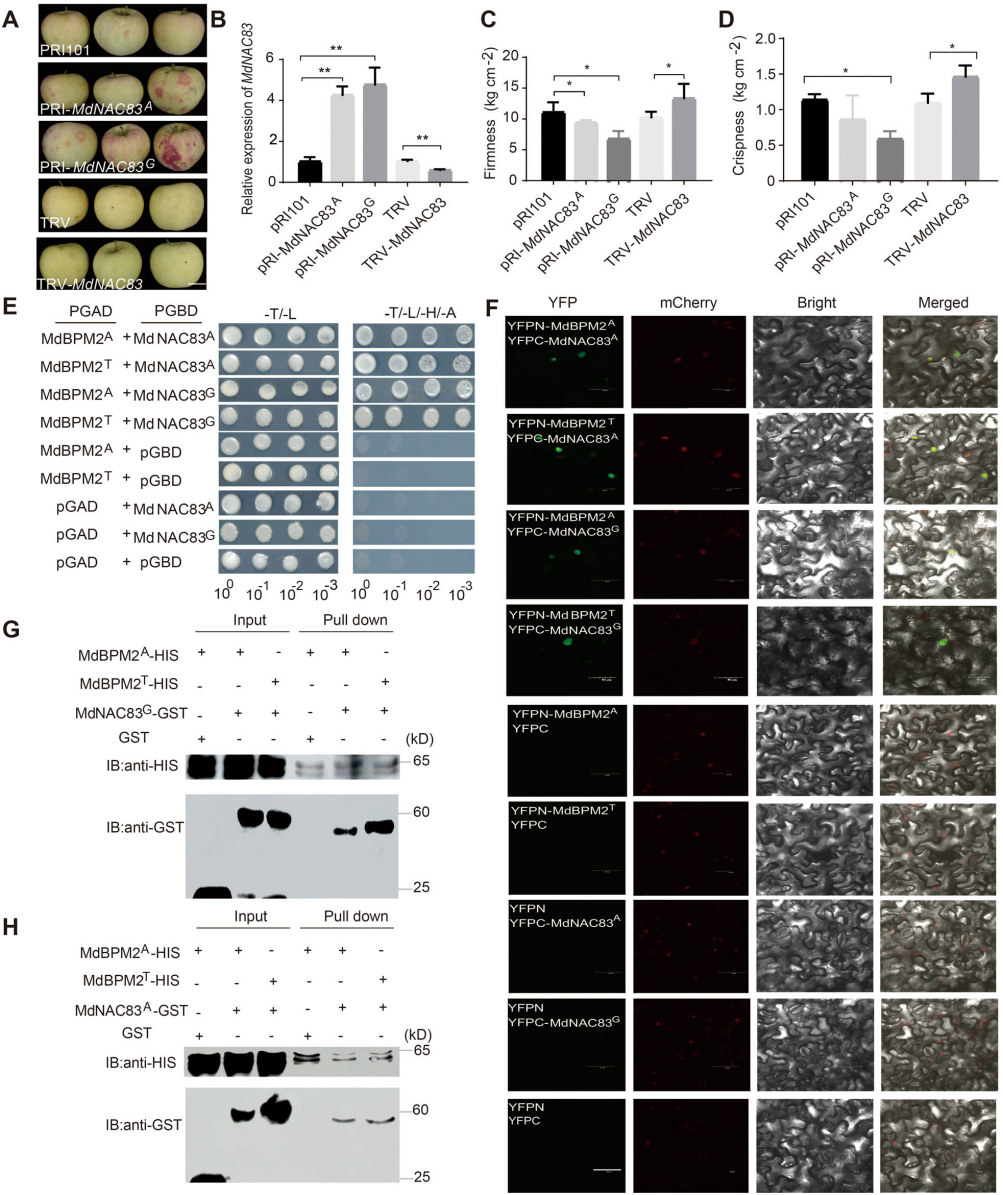
**Transient over‐expression or virus‐induced gene silencing of** ***MdNAC83*** **in ‘Red Fuji’ and validation of the protein–protein interaction between MdBPM2 and MdNAC83** **(A)** Photographs showing appearance phenotype changes after transient transformations in ‘Red Fuji’. Scale bar, 3 mm. **(B)** The expression of *MdNAC83* over‐expression (p*RIMdNAC83*) or silencing (*TRVMdNAC83*) in ‘Red Fuji’. **(C**, **D)** Changes in flesh firmness **(C)** and flesh crispness **(D)** in transiently transformed apples. Asterisks indicate statistical significance (*P < 0.05, **P < 0.01, ***P < 0.001). **(E)** Yeast‐two‐hybrid (Y2H) assay demonstrating the interaction between MdBPM2 and MdNAC83. **(F)** Bimolecular fluorescence complementation (BiFC) analysis showing the *in vivo* interaction between MdBPM2 and MdNAC83 in co‐transformed *Nicotiana benthamiana* leaf epidermal cells. mCherry was used as the nuclear marker. Scale bars, 50 μm. **(G**, **H)** Pull‐down assay showing the *in vitro* interaction of MdBPM2 and MdNAC83. The prokaryotic expression system was used to inducible expression of GST and His fusion proteins. The MdBPM2‐HIS protein was incubated with MdNAC83‐GST or GST and then eluted using a GST purification kit. The eluted proteins were detected with anti‐GST and anti‐HIS antibodies.

To further verify whether the allelic variation in *MdNAC83* CDS might lead to changes in apple flesh texture, transient transformation by over‐expression or virus‐induced gene silencing (VIGS) was performed using unripe fruit of ‘Golden Delicious’ and ‘Red Fuji’, because the genotype of *MdNAC83* SNP388 was GA and GG in ‘Red Fuji’ and ‘Golden Delicious’, respectively (Table S4). The relative expression levels of *MdNAC83* were upregulated in over‐expression or downregulated in VIGS transiently transformed lines three‐ to five‐fold (Figures 3A, B, 4A, B). In transgenic lines of ‘Golden Delicious’ and ‘Red Fuji’, over‐expressing *MdNAC8*3 with SNP388 G allele led to significantly lower flesh firmness and crispness compared with those with the SNP388 A allele, while *MdNAC83* VIGS lines exhibited higher flesh firmness and flesh crispness compared with the transformant with empty TRV vector (Figures 3C, D, 4C, D). In addition, the ethylene production was significantly higher in lines over‐expressing *MdNAC83* with the SNP388 G allele compared with those with the SNP388 A allele, but the *MdNAC83* VIGS lines produced less ethylene than the empty vector transformant of ‘Golden Delicious’ (Figure 3E). Ethylene emission was too low to be quantitatively measured in transgenic “Red Fuji” apples (data not shown). To further explore the function of *MdNAC83*, stably transformed lines of apple calli over‐expressing or with RNAi *MdNAC83* were obtained (Figure 3F). The relative expression of *MdACO1*, *MdMANA3*, and *MdXTH28* was significantly upregulated in lines over‐expressing *MdNAC83* with the SNP388 G allele compared with those over‐expressing the SNP388 A allele, while the expression of *MdACO1*, *MdMANA3*, and *MdXTH28* was inhibited in *MdNAC83* RNAi lines (Figure 3G–I). These data confirmed that *MdNAC83* negatively contributed to apple flesh firmness/crispness retention and that the SNP388 A allele of *MdNAC83* reduced the function.

### Functional validation of allelic variations in *MdBPM2*

To characterize the function of allelic variation in *MdBPM2* CDS affecting apple flesh firmness and crispness, *MdBPM2* was then transiently transformed via *Agrobacterium*‐mediated infiltration or silenced by *pTRV* VIGS in unripe ‘Golden Delicious’ and 'Red Fuji’ (Figure S5A, B, E, F). The *MdBPM2* SNP657 genotype was TT, TA, TT in ‘Red Fuji’, ‘Zisai Pearl’, and ‘Golden Delicious’, respectively (Table S4). No observable changes in skin color were found among transformants (Figure S5A, E). However, flesh firmness and flesh crispness were significantly higher in transiently transformed lines over‐expressing *MdBPM2* with SNP657 A allele than in those with the SNP657 T allele or with the empty vector (Figure S5C, D, G, H). *MdBPM2* VIGS lines showed significantly lower values of flesh firmness and flesh crispness compared with transformants with the empty TRV vector (Figure S5C, D, G, H). The data indicated that the natural variation in *MdBPM2* contributed to the phenotype segregation of flesh firmness and flesh crispness in apple.

### Nonallelic interactions between MdBPM2 and MdNAC83 variants

NAC protein was reported to be negatively regulated by E3 ubiquitin ligase in apple (Li et al., 2022). Here, yeast‐two‐hybrid (Y2H), bimolecular fluorescence complementation (BiFC), and pull‐down assays revealed that the full‐length MdBPM2 interacted with the full‐length MdNAC83 protein, irrespective of their allelic variations (Figure 4E–H).

**Figure 5:**
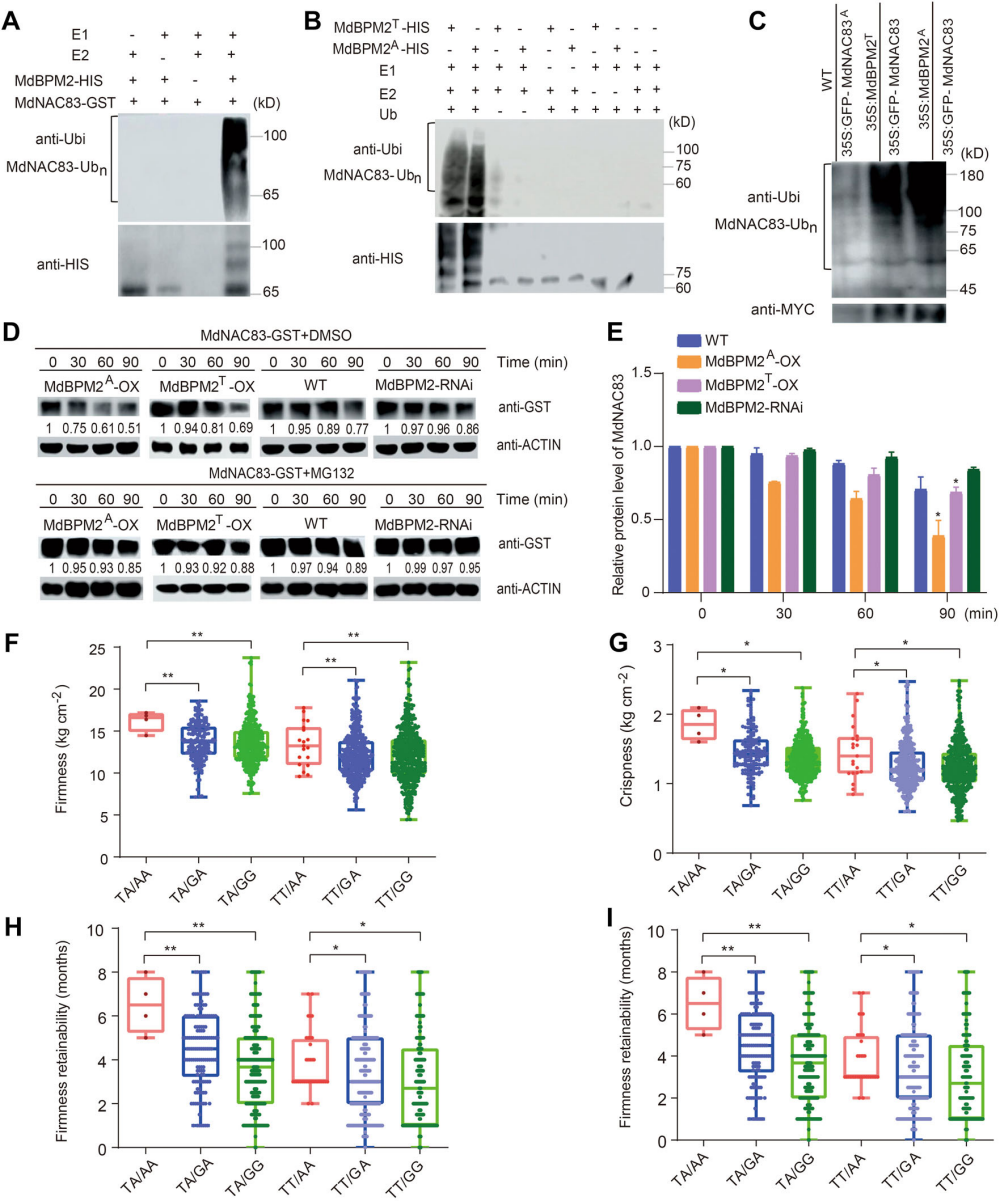
**Nonallelic interaction between** ***MdBPM2*** **and** ***MdNAC83*** **variants** **(A**, **B)** MdBPM2 promoted the ubiquitination of MdNAC83 protein *in vitro*. Purified MdBPM2‐HIS protein and MdNAC83‐GST protein, E1, E2 and Ubi *in vitro* for immunoprecipitation using anti‐His and anti‐Ubi antibodies. **(C)** Ubiquitination of the MdNAC83 protein was detected in 35S::MdNAC83‐GFP and 35S::MdNAC83‐GFP + 35S::MdBPM2‐MYC transgenic apple calli. MdBPM2‐MYC was immunoprecipitated using an anti‐MYC antibody from the transgenic calli. Anti‐Ubi antibody was used to detect the ubiquitination of MdNAC83 protein. **(D)** The ubiquitinated degradation of MdNAC83‐GST protein was differentially promoted by MdBPM2 with SNP657 A or T alleles, which was inhibited by exogenous application of MG132. For dimethyl sulfoxide (DMSO) and MG132 treatments, wild‐type (WT) and MdBPM2‐OX/RNAi apple calli extracts were treated with DMSO or 50 μM MG132 and then incubated with MdNAC83‐GST protein for the indicated time (0, 30, 60, and 90 min). **(E)** Quantification of immunoblot signals. MdNAC83‐GST was detected with anti‐GST antibody. Data are the means ± *SD* of three independent replicates. **(F**–**I)** The joint effects of genotype combinations of MdNAC83 SNP388G/A and MdBPM2 SNP657 T/A on flesh firmness at harvest **(F)**, flesh crispness at harvest **(G)**, flesh firmness retainability **(H)**, and flesh crispness retainability **(I)** using a training population including 1,803 individuals. Asterisks indicate statistical significance (*P < 0.05, **P < 0.01, ***P < 0.001).

To investigate whether MdBPM2 acted as a functional E3 ligase, an *in vitro* ubiquitination assay was conducted. With the presence of E1, E2, and the ubiquitin protein, the ubiquitinated form of MdBPM2‐HIS was detected by both anti‐Ub and anti‐HIS antibodies (Figure 5A). MdNAC83 substrate protein was degraded qualitatively by the MdBPM2 fusion protein, but the ubiquitination level of MdNAC83 by MdBPM2 with the SNP657 A allele fusion protein was slightly higher than that with SNP657 T allele (Figure 5B). These data collectively indicated that allelic variation in MdBPM2 affected its E3 ligase activity.

*In vivo* ubiquitination assays were then performed using two transgenic apple calli: 35S::MdNAC83‐GFP and 35S::MdNAC83‐GFP + 35S::MdBPM2‐MYC. The abundance of polyubiquitinated MdNAC83‐GFP protein in 35S::MdNAC83‐GFP + 35S::MdBPM2^A^‐MYC apple calli was higher than that in 35S::MdNAC83‐GFP and 35S::MdNAC83‐GFP + 35S::MdBPM2^T^‐MYC (Figure 5C). The data indicated that the MdBPM2 fusion protein with SNP657 A allele had a higher ubiquitination ability than the one with the SNP T allele *in vivo*.

Furthermore, cell‐free degradation assays of the prokaryotic‐expressed and purified MdNAC83‐GST fusion proteins were conducted using protein samples extracted from wild‐type (WT), 35S::MdBPM2^A/T^, and 35S::anti‐MdBPM2 transgenic apple calli. The MdNAC83‐GST protein was degraded more significantly in the protein extract of the 35S::MdBPM2^A/T^ than in that of *MdBPM2* RNAi lines and the WT. The MdBPM2 fusion protein with the SNP657 A allele had a higher ubiquitination ability than with the SNP T allele, whereas the protein was more stable in the protein extract of 35S::anti‐MdBPM2 compared with the WT (Figure 5D, E). However, the abundance of MdNAC83‐GST protein was unchanged in the apple calli in the presence of the proteasome inhibitor MG132 (Figure 5D). Overall, these results suggested that the MdNAC83 protein was degraded by MdBPM2 in a 26S proteasome‐dependent manner.

To show the effect of nonallelic interaction between *MdBPM2* and *MdNAC83* variants on the phenotype of apple fruit storability, the joint effect of genotype combinations was estimated using a training population including 1,803 individuals. Significant differences were observed in the joint effects of genotype combinations of *MdNAC83* SNP388 G/A and *MdBPM2* SNP657 T/A on FF, FC, FFR, and FCR (Figure 5F–I). The genotype combination *MdBPM2* SNP657 TA/*MdNAC83* SNP388 AA exerted the highest joint effects on FF, FC, FFR, and FCR, respectively (Figure 5F–I). By contrast, the genotype combination of *MdBPM2* SNP657 TT/*MdNAC83* SNP388 GG exhibited the lowest joint effects on FF, FC, FFR, and FCR, respectively (Figure 5F–I). In addition, the genotype combination of *MdBPM2* SNP657 TT/*MdNAC83* SNP388 GG showed the highest joint effects on fruit ethylene production after 120 days of cold storage using 36 randomly chosen hybrid lines (Figure S6A).

### Functional validation of allelic variations in *MdRGLG3*

The genotype of *MdRGLG3* SNP167 was CG and CC in ‘Red Fuji’ and ‘Golden Delicious’, respectively (Table S4). Transiently transformed 'Golden Delicious' and 'Red Fuji' apples over‐expressing *MdRGLG3* with SNP167 G allele exhibited significantly higher flesh firmness and flesh crispness than those with the SNP167 C allele (Figure S7). Both lower flesh firmness and flesh crispness were observed in *MdRGLG3* VIGS lines compared with that in the TRV empty lines (Figure S7). These data demonstrated that allelic variation in *MdRGLG3* SNP167 C/G could affect fruit flesh firmness and flesh crispness.

### Nonallelic interactions between MdRGLG3 and MdNAC83 variants

**Figure 6:**
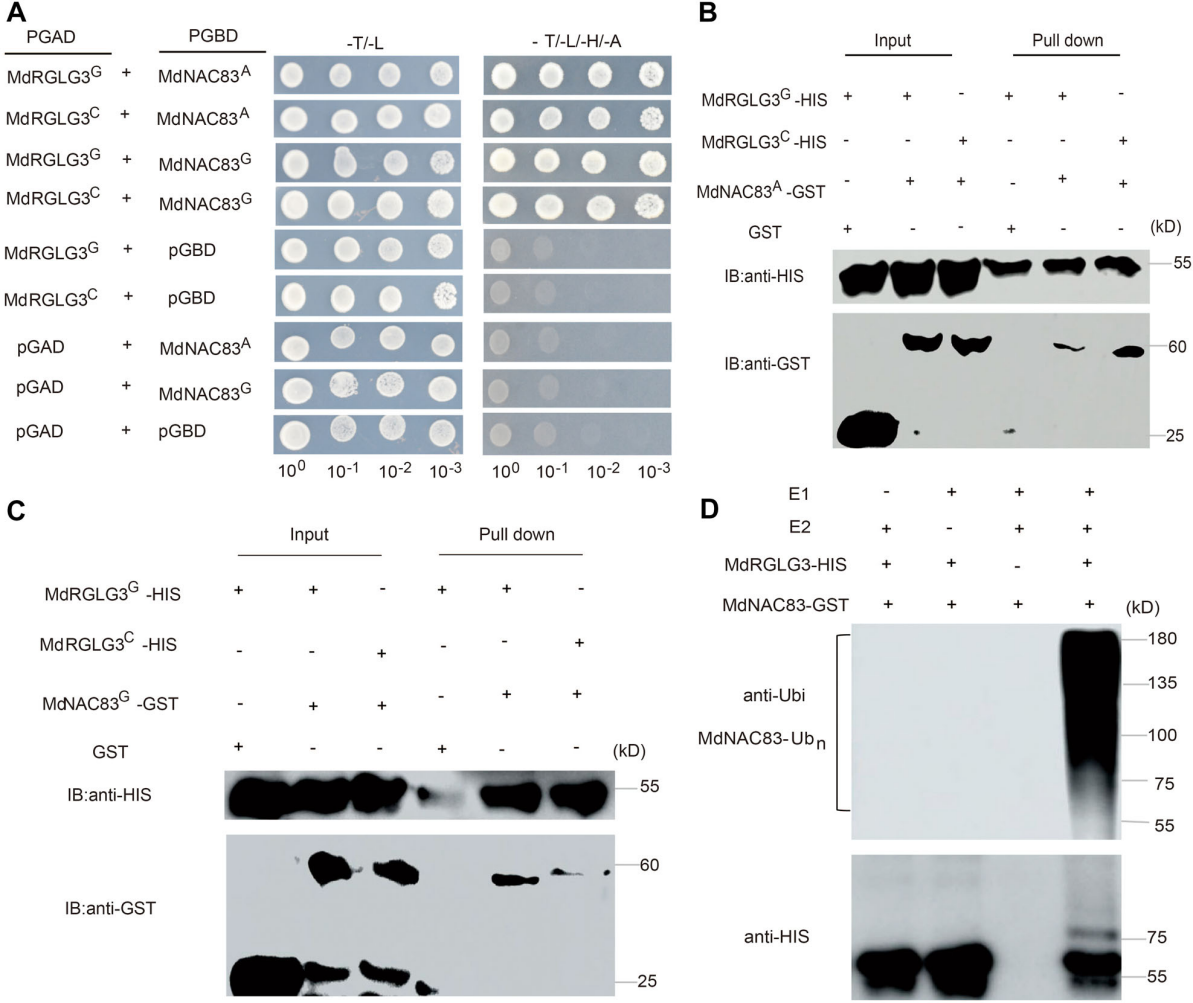
Nonallelic interaction between MdRGLG3 and MdNAC83 **(A)** Yeast‐two‐hybrid assay showing the protein–protein interaction between variants of MdRGLG3 and MdNAC83. **(B**, **C)** Pull‐down assay showing the interaction between MdRGLG3 and MdNAC83. The MdRGLG3‐HIS protein was incubated with MdNAC83‐GST or GST and then eluted using a GST purification kit, with the anti‐GST antibody and anti‐HIS antibody. GST alone was used as the control. **(D)** Immunoprecipitation assay demonstrating the *in vitro* ubiquitination of MdNAC83 by MdRGLG3. The MdRGLG3‐HIS and MdNAC83‐GST purified protein, E1, E2, and Ubi at 30°C for immunoprecipitation, using anti‐Ubi and anti‐HIS antibodies.

**Figure 7:**
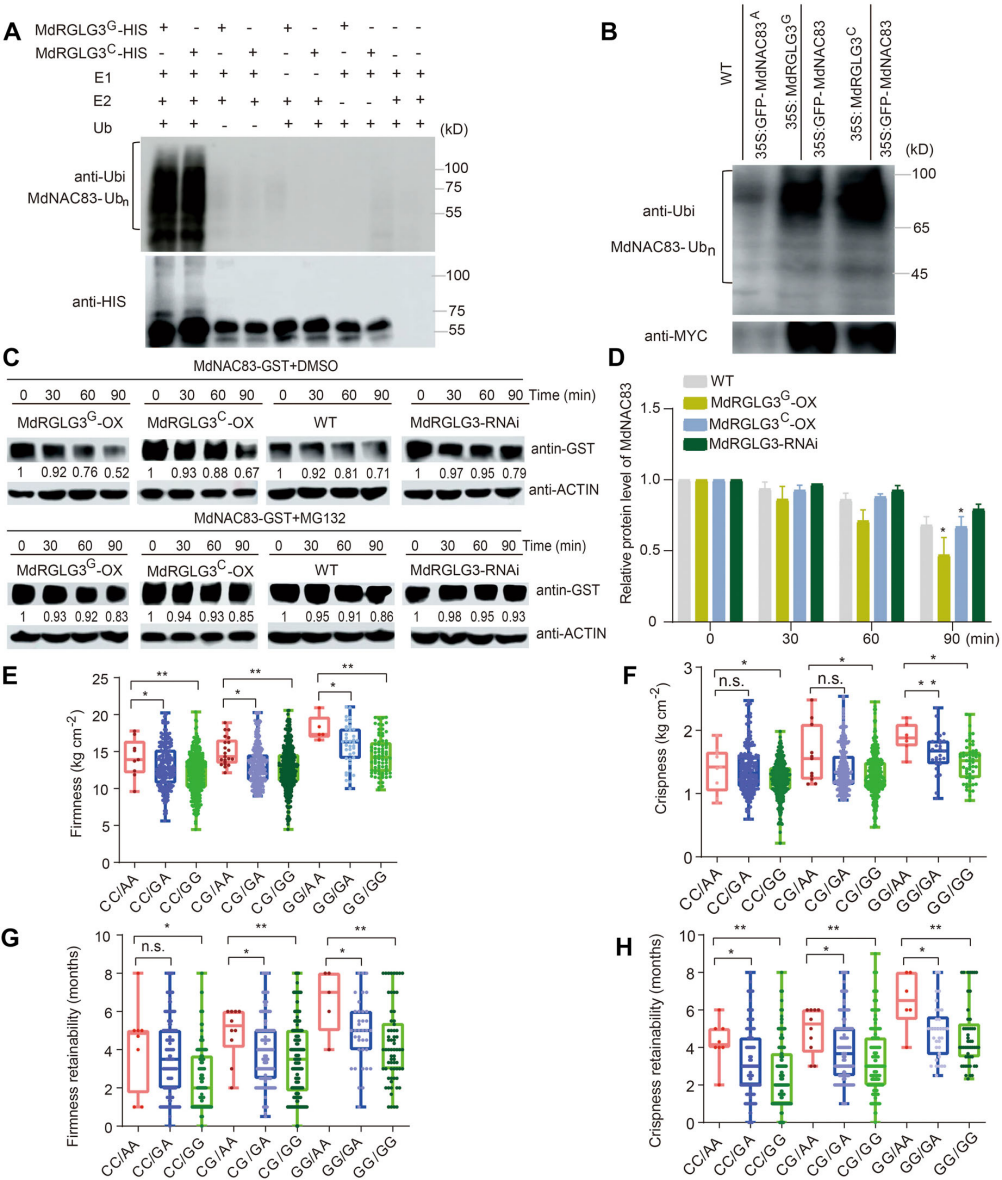
Validation of the interaction between MdRGLG3 and MdNAC83 by protein ubiquitination and joint effects of genotype combinations **(A)** MdRGLG3 promoted the ubiquitination of MdNAC83 protein *in vitro*. **(B)** Ubiquitination assays *in vivo* using 35S::MdNAC83‐GFP and 35S::MdNAC83‐GFP + 35S::MdRGLG3‐MYC. anti‐MYC and anti‐Ubi antibodies were used to examine immunoprecipitation. **(C)** The ubiquitination ability of MdRGLG3 with SNP167 G allele on MdNAC83‐GST protein was higher than that with the SNP167 C allele, but the ubiquitinated degradation of MdNAC83‐GST protein was inhibited by exogenous application of MG132. Wild‐type and MdRGLG3‐OX/RNAi apple calli extracts were treated with DMSO or 50 μM MG132 and then incubated with MdNAC83–GST protein for the indicated time (0, 30, 60, and 90 min). **(D)** Quantification of immunoblot signals. MdNAC83‐GST was detected with anti‐GST antibody. Data are the means ± *SD* of three independent experiments. **(E–H)** The joint effects of genotype combinations of MdNAC83 SNP388 G/A and MdRGLG3 SNP167 C/G on flesh firmness at harvest **(E)**, flesh crispness at harvest **(F)**, flesh firmness retainability **(G)**, and flesh crispness retainability **(H)** of the training population. Asterisks indicate statistical significance (*P < 0.05, **P < 0.01, ***P < 0.001).

MdRGLG3 is an E3 ligase that promotes the degradation of target proteins (Yin et al., 2007; Wu et al., 2016). The *in vitro* interaction between MdRGLG3 and MdNAC83 was identified through Y2H and was confirmed by pull‐down assay (Figure 6A–C). The allelic variations in either MdRGLG3 or MdNAC83 did not interfere with the interaction between them (Figure 6B, C). *In vitro* immunoprecipitation assays showed that higher amounts of high‐molecular mass forms of MdNAC83 protein were detected in the immunoprecipitated mixture, indicating that MdRGLG3 can ubiquitinate MdNAC83 protein (Figure 6D). MdRGLG3 also exhibited self‐ubiquitination activity (Figure S8). The ubiquitination degree of MdNAC83 was slightly higher by MdRGLG3 fusion protein with the SNP167 G allele than with the SNP167 C allele (Figure 7A). To verify the ubiquitination of MdNAC83 protein by MdRGLG3 *in vivo*, we obtained double transgenic apple calli 35S::MdNAC83‐GFP + 35S::MdRGLG3^G^‐MYC and 35S::MdNAC83‐GFP + 35S::MdRGLG3^C^‐MYC from the single transgenic calli 35S::MdNAC83‐GFP. The abundance of polyubiquitinated MdNAC83‐GFP protein in 35S::MdNAC83‐GFP + 35S::MdRGLG3^G^‐MYC was much higher than that in single 35S::MdNAC83‐GFP and 35S::MdNAC83‐GFP + 35S::MdRGLG3^C^‐MYC (Figure 7B). Cell‐free degradation assays revealed that the degradation rate of MdNAC83‐GST was higher in the MdRGLG3^G/C^‐OX extract and lower in the MdRGLG3‐anti extract, compared with the WT, and the MdRGLG3 fusion protein with the SNP167 G allele exhibited higher ubiquitination ability on MdNAC83 protein than with the SNP C allele (Figure 7C, D). However, the degradation of MdNAC83‐GST was restrained by the proteasome inhibitor MG132 (Figure 7C), suggesting that MdRGLG3 ubiquitinated MdNAC83 protein through the 26S proteasome pathway. In summary, the MdRGLG3 fusion protein with the SNP167 G allele had a higher ability than that with the SNP167 C allele to ubiquitinate the MdNAC83 protein both *in vitro* and *in vivo*.

The nonallelic interaction between *MdRGLG3* and *MdNAC83* variations was also confirmed by the significant differences in the joint effects of genotype combinations on FF, FC, FFR, and FCR of the training population (Figure 7E–H). The highest joint effects on FF, FC, FFR, and FCR were estimated in genotype combination *MdRGLG3* SNP167 GG/*MdNAC83* SNP388 AA, while the *MdRGLG3* SNP167 CC/*MdNAC83* SNP388 GG genotype combination exhibited the lowest joint effects on FF, FC, FFR, and FCR, respectively (Figure 7E–H). Additionally, significant differences in the joint effect on fruit ethylene production after 120 days of cold storage were observed between the *MdNAC83* and *MdRGLG3* variants, while the highest joint effects were exerted by the genotype combination of *MdRGLG3* SNP167 CC/*MdNAC83* SNP388 GG (Figure S6B).

### Development of functional markers and their application in GAP for fruit storability

GenoBaits probes were designed flanking the functional variations of *MdBPM2*, *MdNAC83*, and *MdRGLG3* in this study, as well as those of *MdERF3*, *MdERF118*, *MdPAE10*, *MdbHLH25*, and *MdWDR5A*, which we had previously reported (Wu et al., 2021a; 2021b; Yang et al., 2022). We found that all the functional markers exhibited partial dominant allelic interaction according to the genotype effect estimates, while a few linkage markers showed dominant (e.g., Chr16_38574538 on FF, Chr15_29178910 on FC, etc.) or additive (e.g., Chr15_16181140 on FC, Chr16_38573461 on FC, etc.) allelic interaction (Table S5). The marker effects varied as 0.21–40.25 kg/cm^2^, 0.01–1.21 kg/cm^2^, 0.07–4.42 months, and 0.05–5.06 months on FF, FC, FFR, and FCR, respectively (Table S5).

**Figure 8:**
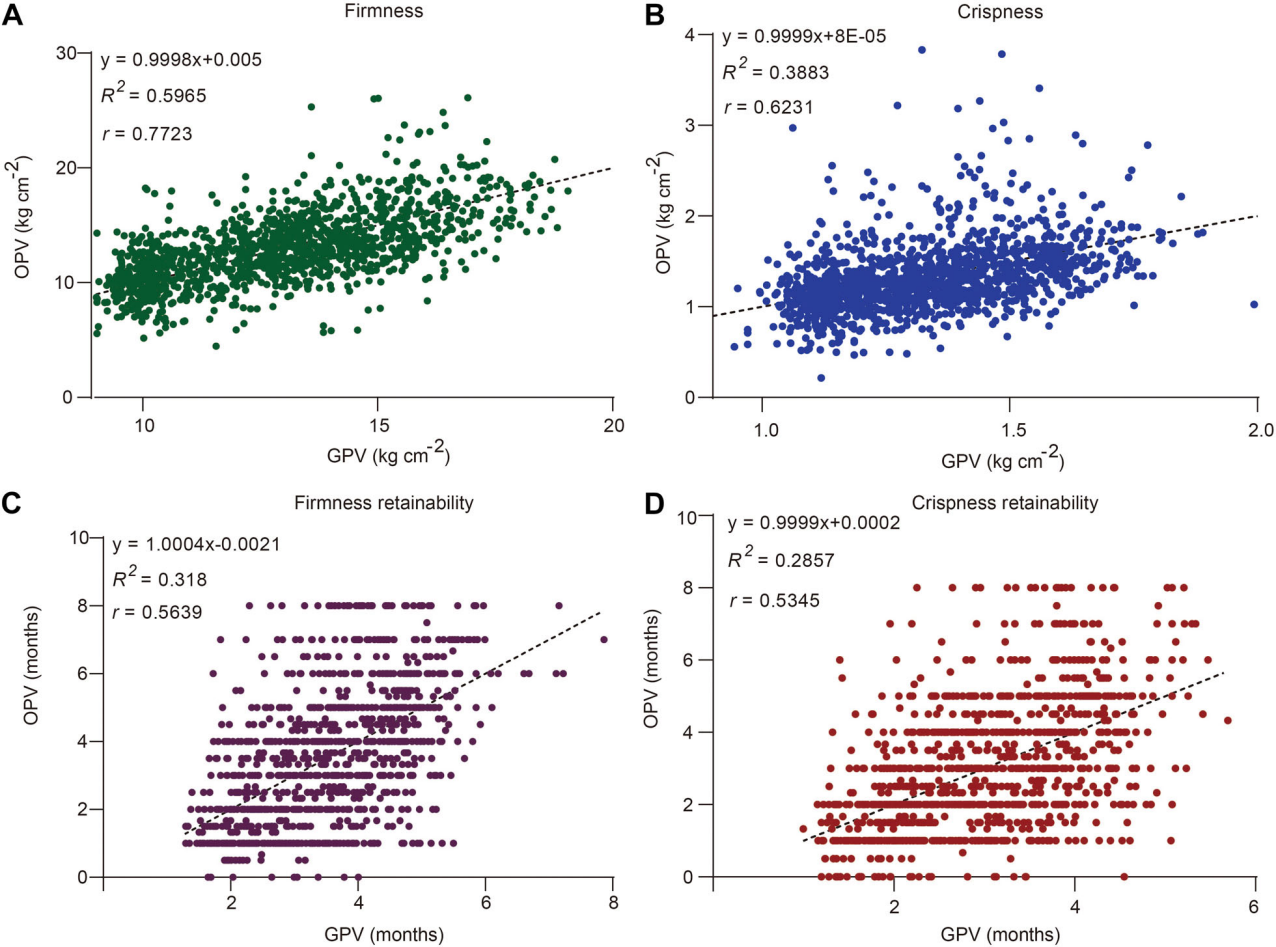
Prediction accuracy of non‐additive genomics‐assisted prediction models for apple storability **(A**–**D)** The prediction accuracy of non‐additive genomics‐assisted prediction models for flesh firmness at harvest **(A)**, flesh crispness at harvest **(B)**, flesh firmness retainability **(C)**, and flesh crispness retainability **(D)** using three groups of joint effects of genotype combinations of functional markers as fixed effects. Linear regression is shown between genotype predicted value (GPV) and observed phenotype value (OPV).

Addition of QTL‐derived markers to a GS model led to an increased prediction accuracy in both animals and plants (Liabeuf et al., 2018; Ma et al., 2019). In this study, 13 GenoBaits markers spanning or linked to *MdBPM2* SNP657, *MdNAC83* Del216, SNP388, and SNP934; *MdRGLG3* SNP167; *MdERF3* Del8; *MdERF118* Del3; *MdPAE10* Del379; *MdbHLH25* SNP1, SNP2, and SNP4/5; and *MdWDR5A* SNPi and SNPii were added to the marker array panel (Table S5). By the addition of these markers, the prediction accuracy of the additive GAP models was 0.7638, 0.6002, 0.5512, and 0.5201 for FF, FC, FFR, and FCR, respectively (Figure S9). Due to the non‐additive allelic or nonallelic interactions among the functional variations, three subsets of joint effects of interacting functional markers were estimated and applied in the GAP models (Table S7). The prediction accuracy of the non‐additive GAP models was further increased to 0.7723, 0.6231, 0.5639, and 0.5345 for FF, FC, FFR, and FCR, respectively (Figure 8A–D).

### Genetic composition and structure analysis of markers for fruit storability

To explore the origin and the evolution of genetic variations in fruit storability‐related genes, genetic composition and structure analysis were carried out using genotype data of 257 markers associated with FF, FC, FFR, and FCR in 612 accessions from six *Malus* species. Genetic structure analysis (http://pophelper.com) indicated that *K* = 4 was a sensible modeling choice. Gene introgression into *M. domestica* cultivars was found from three sources: *M. sieversii* (dark blue), *M. baccata* (yellow), and an unknown species (red) (Figure S10). Gene introgression was also detected from *M. sieversii* (dark blue) and *M. baccata* (yellow) into traditional Chinese domesticated apple accessions, *M. asiatica* and *M. robusta* (Table S8). Genetic composition analysis was performed using 18 markers with large genotype effects on FCR (Table S8). High frequencies of homozygous genotypes of seven markers were found in both *M. sieversii* and *M. baccata* (Table S8). Two of the seven markers (Chr15_40410846 and Chr16_ 3059544, with green mark) were fruit storability positive and the other five (Chr02_10434413, Chr03_25532731, Chr10_40756571, Chr16_38546603, and Chr16_38574583, with yellow mark) were negative to fruit storability (Table S8). An obvious decrease in the frequency of both fruit storability‐positive and ‐negative homozygous genotypes of these seven markers were found in *M. domestica* cultivars (Table S8). The frequencies of fruit storability‐positive homozygous genotype of seven markers (with dark blue mark) were higher in *M. baccata* than that in *M. sieversii*, including Chr03_30739052, *MdBPM2* SNP657 (Chr03_35391111), *MdDof3.5* (Chr14_3790454), *MdPAE10* (Chr16_10069605), *MdXTH28* (Chr16_1074586), *MdNAC83* Del216 (Chr16_9126219_9126434), and *MdNAC83* SNP934 (Chr16_9127269) (Table S8). The GG genotype of *MdRGLG3* SNP167 (Chr16_38704799) was only found in *M. domestica* and *M. sieversii*. The frequency of the AA genotype of Chr03_30739052 was even higher in *M. asiatica* than in *M. baccata*, while a relatively higher frequency of fruit storability‐positive genotypes of *MdDof3.5* (Chr14_3790454), *MdXTH28* (Chr16_1074586), *MdNAC83* Del216 (Chr16_9126219_9126434), and *MdNAC83* SNP934 (Chr16_9127269) was also detected in *M. prunifolia* and *M. robusta* (Table S8). These data implied a different domestication route between the *M. domestica* and Chinese domesticated apple cultivars.

## DISCUSSION

**Figure 9:**
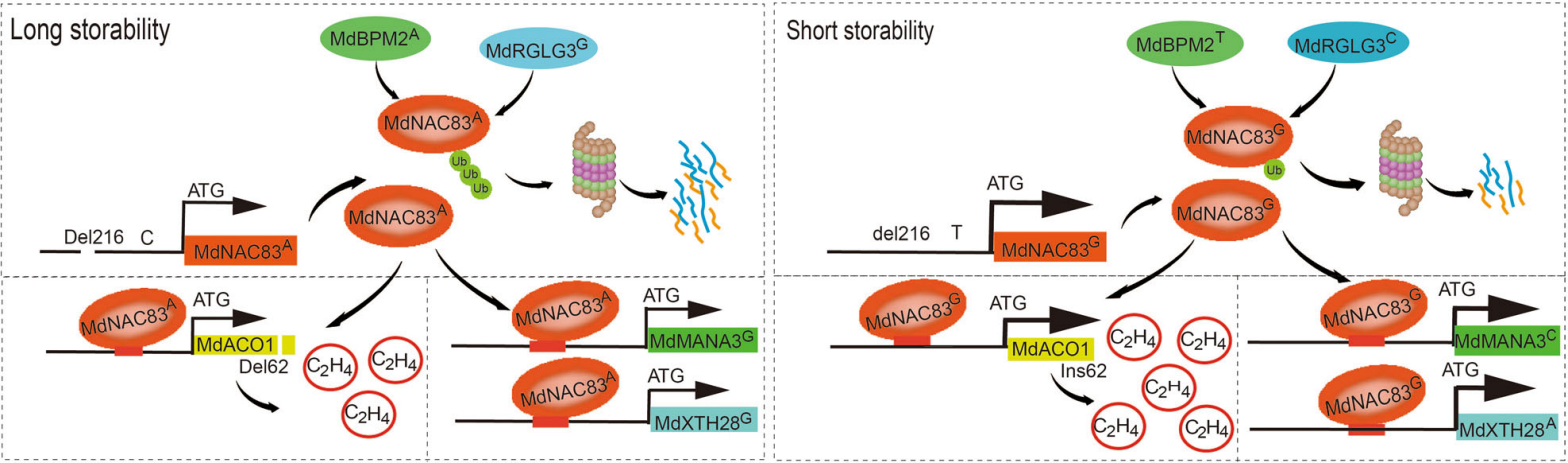
Genetic variation network controlling apple fruit storability characterized by flesh firmness and crispness retainability The two E3 ligases, MdBPM2 and MdRGLG3, ubiquitinate MdNAC83 causing its degradation. MdBPM2 SNP657 A and MdRGLG3 SNP167 G exhibit stronger ubiquitinating activity. MdNAC83 Del216 and SNP934 C at the promoter lead to the lower transcription activity of *MdNAC83*. MdNAC83 SNP388 A at the coding sequence show lower activity to activate the downstream target genes, *MdACO1*, *MdMANA3* and *MdXTH28*, while MdACO1 Del62, MdMANA3 SNP546 G, and MdXTH28 SNP591 G cause reduction in their enzyme activity. All these variations together lead to longer cold storability of apple fruit (left). Conversely, MdBPM2 SNP657 T and MdRGLG3 SNP167 C exert stronger ubiquitinating activity on MdNAC83 ubiquitinated degradation. MdNAC83 del216 and SNP934 T at the promoter cause higher transcription activity of *MdNAC83*. MdNAC83 with SNP388 G at the coding sequence has higher activity to promote the expression of *MdACO1*, *MdMANA3* and *MdXTH28*. MdACO1 Ins62, MdMANA3 SNP546 C, and MdXTH28 SNP591 A have higher enzyme activity. All these variations contribute to shorter storability of apple fruit (right).

Apple fruit storability is a complicated quantitative trait, which is proposed to be controlled by a large number of genetic variations; therefore, the empirical predictive power of fewer functional markers was limited (Nybom et al., 2013). We found in this study that a genetic variation network constituted of eight Mendelian variations in six genes contributed to the phenotype variations in apple FFR and FCR (Figure 9). The functional variations in *MdNAC83* (Del216, SNP934 T/C, and SNP388 G/A), *MdBPM2* (SNP657 T/A), and *MdRGLG3* (SNP167 C/G) were identified and validated in this study. Variations in *MdMANA3* and *MdXTH28* exerted considerable genotype effects on apple fruit storability, but the function of these variations has not yet been validated. The contribution of each genetic variation in the network to the phenotype segregation and the allelic or nonallelic interactions was quantified (Figure 9; Table S5).

Ethylene production and signaling pathway are well understood in the network controlling fruit storability, functional variations in *MdACS1*, *MdACO1*, *MdERF3*, and *MdERF118* have been previously validated (Harada et al., 2000; Costa et al., 2005; Wu et al., 2021b). An SNP in the EAR motif in the coding region of *MdERF4* caused a reduction in the protein–protein interaction between MdERF4 and MdTPL4, which resulted in reduced repression of *MdERF3* expression and subsequently reduced in *MdACO1* expression, and ethylene production, but increased in apple fruit firmness (Hu et al., 2020). Variations in *MdACO1* and *MdERF118* exerted a relatively high genotype effect on FF, FC, FFR and FCR (Table S5). Additionally, allelic variations in cell wall metabolism genes downstream of ethylene signals played more important roles in fruit storability (Costa et al., 2008; 2010). High genotype effects were estimated for markers designed in *MdPAE10*, *MdPME21*, *MdXTH28*, and *MdMANA3* (Table S5).

Most importantly, genetic variations in the regulatory genes upstream of ethylene and cell wall pathways contributed more effect to the phenotype variations of fruit storability. NAC family transcription factors have been reported to control fruit ripening and fruit firmness (Forlani et al., 2021; Migicovsky et al., 2021). An SNP in CmNAC‐NOR contributes to fruit ripening by regulating the expression of ethylene and ABA biosynthetic genes, color formation genes, and sugar biosynthetic genes in climacteric melon (Wang et al., 2022). Variations in *MdNAC18* were reported to affect apple fruit firmness (Larsen et al., 2019). We previously identified and validated that the functional variation SNP517 T/A and SNP958 A/G played key roles in regulating apple fruit ripening date (Wen et al., 2024). In this study, the genotype effects of *MdNAC18* SNP517 AA, TA, and TT on FCR were estimated as −0.19, 0.07, and 0.54 months, respectively (Table S5). These data indicated that *MdNAC18* SNP517 contributed to apple post‐harvest storability although the genotype effects were relatively lower than that of *MdNAC83* (Table S5). It is easy to understand the effects of variations of *MdNAC18* on FCR because *MdNAC18* positively regulates the expression of ethylene biosynthesis gene *MdACO1–2* directly and *MdACS1* indirectly mediated by *MdARF5* (Wen et al., 2024). In this study, we found that *MdNAC83* acted as a central master, which integrated genetic variations in both ethylene‐dependent and ethylene‐independent pathways, participating in the genetic control of apple fruit storability. *MdNAC83* participated in the regulation of fruit storability via an ethylene‐dependent model by directly binding to the promoter of *MdACO1* and active gene expression, but also in an ethylene‐independent manner by regulating the expression of cell wall metabolism genes such as *MdXTH28* and *MdMANA3* (Figures 2B–E, 3G–I and 9).

Transient transformation of apples confirmed that *MdNAC83* acts to negatively regulate apple fruit storability. Genetic variations at both the promoter (such as Del216) and the CDS (such as SNP388 G/A) of *MdNAC83* affected its function. We found that the SNP388 A allele reduced the transcription factor activity of *MdNAC83* on target genes like *MdACO1*, *MdMANA3*, and *MdXTH28*, which exerted positive effects on fruit storability. The expression of *MdNAC83* containing the Del216 allele at the promoter was higher than that carrying the del216 allele, which was inconsistent with the results that the genotype effect of *MdNAC83* Del216 on fruit storability was positive, the mechanism of this discrepancy is still unclear.

Ethylene production was closely associated with the allelic variations in *MdNAC83* (Figure 3E). Protein ubiquitination plays an important role in regulating protein activity in eukaryotes, with significant impacts on plant growth and development (Vierstra, 2003; Shi et al., 2018; Hu et al., 2019). Ubiquitination of MdNAC83 by MdBPM2 and MdRGLG3 was involved in the regulation of apple fruit storability. A novel post‐translational modification fine‐tuning module was formed by the genetic variations in not only the substrate protein MdNAC83, but also in the interacting factors MdBPM2 and MdRGLG3 (Figure 9). Both MdBPM2 and MdRGLG3 E3 are ubiquitin ligases belonging to two distinct subfamilies (Figure S11A), however, MdNAC83 was the common target protein of the two E3 ligases MdBPM2 and MdRGLG3 in regulating apple fruit storability (Figure 9). The marker effects of *MdRGLG3* SNP167 on FF, FC, FFR, and FCR were relatively higher than those of *MdBPM2* SNP657 (Figure S3). The expression levels (in FPKM) of *MdRGLG3* were higher than that of *MdBPM2* during apple fruit storage based on the previous RNA‐seq data (Figure S11B, C) (Wu et al., 2021b). Variations in *MdbHLH25* and *MdWDR5A* controlled fruit storability intermediated by ethylene synthesis gene *MdACS1* and *MdACO1* (Yang et al., 2022). *MdDof5.3* and *MdRAL1* participated in the regulation of apple fruit storability via ethylene signaling genes *MdERF3* and *MdERF118* (Wu et al., 2021b). Genetic variations in the *MdNAC83/MdBPM2/MdRGLG3* module affected apple fruit softening by both directly regulating cell wall catabolism genes such as *MdMANA3* and *MdXTH28* and indirectly regulating cell wall degradation via the ethylene biosynthesis gene *MdACO1*, which is quite like the *MdERF3/MdERF118* regulatory pathway (Wu et al., 2021b). However, the joint effects of the genotype combinations of variations in *MdNAC83/MdBPM2/MdRGLG3* on FF, FC, FFR, and FCR deviated to 6.2 kg/cm^2^, 0.38 kg/cm^2^, 3.04 months, and 3.31 months, respectively (Table S9), which were larger than those of *MdERF3/MdERF118* (Wu et al., 2021b). The genetic variation network controlling fruit storability functioned at multifaceted nonallelic interaction levels, i.e., phytohormonal, transcriptional, and post‐translational levels.

The complexity in the genetic control of a quantitative trait is also attributed to the fact that a QTL may harbor multiple functional variations, even in one gene region. In apple rootstock, two functional SNPs at the promoter of *MdLAZY1* caused wider phenotype segregation of root growth angle in leafy cuttings (Zheng et al., 2020a). Within the QTL F16.1 for FFR and FCR, three and two SNPs were identified and validated to be functional variations in two interacting genes, *MdbHLH25* and *MdWDR5A*, respectively (Yang et al., 2022). Both SNP517 T/A and SNP958 G/A at *MdNAC18* CDS were functional variations affecting apple fruit ripening date (Wen et al., 2024). We found in this study that the three functional variations in *MdNAC83* extensively broadened the spectrum of phenotype segregation in FF, FC, FFR, and FCR (Figure S2A–D).

Diagnostic markers are desirable for molecular breeders to improve the prediction accuracy of GS models (Leng et al., 2017; Mason et al., 2018). By the addition of the diagnostic markers developed from the above‐mentioned functional variations to the GAP models, the prediction accuracy of the additive GAP models for fruit storability traits increased compared with the previous model with linkage markers (Figure S9). However, the non‐additive effects contribute a large proportion of genetic effects in outbred plants like fruit trees (Zheng et al., 2020b; Shen et al., 2022). Dominant or partially dominant allelic effects were common in apple (Shen et al., 2022). Non‐additive effects should be taken into full consideration in GS models (Muranty et al., 2015; Di Guardo et al., 2017). The joint effects of genotype combinations of markers developed in the interacting genes were input to the GAP models, thus, the allelic dominance and nonallelic epistasis were included in the models (Zheng et al., 2020b; Wen et al., 2024). The prediction accuracy of the non‐additive GAP models for apple FF, FC, FFR, and FCR in this study was further improved than the additive ones (Figure 8). The predictability of these models was equivalent to or better than that of pure GS, the accuracy of which was 0.08*–*0.45 for changes in fruit firmness or 0.6–0.7 for fruit firmness and crispness in apple (McClure et al., 2018; Kumar et al., 2020). The relatively low heritability is one of the constraints to further improve the prediction accuracy (Wu et al., 2021b).

The positive genetic variations were pyramided during the lengthy natural evolution and inadvertant selection. *M. sieversii* is believed to be the common ancestral species of *M. domestica* and ancient Chinese landrace apple cultivars (Velasco et al., 2010; Duan et al., 2017). Using 257 markers associated with apple fruit storability in this study, considerable gene introgression into *M. domestica* cultivars was detected from *M. sieversii*, *M. baccata*, and an undetermined ancestry species (Figure S10). The unknown ancestor should be *M. sylvestris* because gene introgression from European crabapple *M. sylvestris* and/or *M. orientalis* into *M. domestica* was proposed in several studies (Cornille et al., 2014; Duan et al., 2017; Sun et al., 2021). Conversely, in *M. asiatica* and *M. robusta*, genetic introgression was also found from *M. sieversii* and *M. baccata*, which is consistent with the hypothesis of bi‐directional domestication of cultivated apple (Duan et al., 2017). The frequency of the storability‐positive homozygous genotype in *M. prunifolia* and *M. robusta* was quite similar to *M. baccata* in this study, which supports that the Chinese native species, such as *M. prunifolia* and *M. robusta* are descendants of *M. baccata* (Duan et al., 2017).

During the process of domestication and improvement, natural and anthropogenic selection signatures have been detected for fruit size and acidity in *M. domestica* cultivars by pyramiding of multiple genes with minor effects (Liao et al., 2021). The higher frequency of the storability‐positive homozygous genotype was found in *M. domestica*, such as *MdACO1* (CC, Chr10_40756571), *MdMANA3* (AA, Chr02_10434413), and *MdSGS3* (CC, Chr03_31718792), which implied the footprint of selection events for fruit storage or shelf‐life during improvement (Nybom et al., 2012). Conversely, a high frequency of storability‐positive genetic variations was found in the close relative species such as *M. baccata*, *M. prunifolia*, and *M. robusta*, which brings us a great reservoir of elite variations for future genetic improvement and genome editing in *M. domestica* cultivars.

## MATERIALS AND METHODS

### Plant materials

In total, 612 *Malus* germplasm accessions and 1,191 hybrid plants from ‘Zisai Pearl’ × ‘Red Fuji’, ‘Zisai Pearl’ × ‘Golden Delicious’, and ‘Jonathan’ × ‘Golden Delicious’ were used as the training population. ‘Zisai Pearl’ is a Chinese landrace cultivar that belongs to *M. asiatica* Nakai, ‘Red Fuji’, ‘Jonathan’, and ‘Golden Delicious’ are *M. domestica* Borkh. cultivars.

Three intact apples were harvested at optimum maturity based on the ground color and starch degradation (at 6 on a scale of 1–10) (Blanpied and Silsby, 1992). Fruit samples for laboratory assay were collected, immediately frozen in liquid nitrogen, and stored at −80°C until use.

The apple calli used in this study were induced from the mesocarp of the unripe ‘Orin’ apple fruit (*M. domestica* Borkh.), these calli were sub‐cultured every three weeks before use.

‘Golden Delicious’ and ‘Red Fuji’ fruit for transient transformation were picked at 120 and 170 days after flower blossom (DAFB), respectively.

### Phenotyping for fruit ethylene production and flesh firmness/crispness

Apple fruit cold storage and phenotyping of FF, FC, FFR, and FCR were performed following the previous strategy (Wu et al., 2021b). Phenotype data of FF and FC were collected in the years 2014–2022 and the phenotype data of FFR and FCR were collected in 2016, 2017, 2021, and 2022. Flesh firmness and crispness were measured with a computer‐driven texture analyzer TAXT (Stable Micro System, Godalming, UK) (Costa et al., 2011; 2012; Wu et al., 2021a; 2021b). The penetrating probe diameter was 0.2 cm, and the penetration depth was 0.5 cm (Wu et al., 2021b). During the cold storage, apples were sampled each month with at least three apples as biological replicates and three measurements per fruit as technical replicates. Retainability of flesh firmness and crispness was characterized by the maximum time (months as a unit) until which the apples maintained acceptable flesh firmness (≥ 7.0 kg/cm^2^) and crispness (≥ 0.7 kg/cm^2^), respectively (Costa et al., 2010; Nybom et al., 2012; Wu et al., 2021b).

Ethylene emission was measured by gas chromatography. Each fruit was weighed and enclosed in a gas‐tight container and kept for 3 h at room temperature. After which one milliliter of gas was sampled from the headspace in the container using a BD syringe (No. 309602, BD, Franklin Lakes, NJ, USA). The ethylene concentration of gas samples was measured with a gas chromatograph (HP 5890 series II, Hewlett‐Packard, Palo Alto, CA, USA) equipped with a flame ionization detector. The fruit ethylene production was calculated as described previously (Dougherty et al., 2018).

### QTL mapping and narrowing down of the QTL regions

To show the robustness of QTL regions that we previously identified via BSATOS (Shen et al., 2022), MapQTL v6.0 software was used here to call QTLs for apple FF, FC, FFR, and FCR using linkage maps that we previously published (van Ooijen et al., 2009; Tan et al., 2017). The mapping population included 251 hybrid plants from ‘Zisai Pearl’ × ‘Red Fuji’, while 640 microsatellite markers and 490 SNP markers were used for linkage map construction (Tan et al., 2017). The overlapping QTLs between the two mapping strategies, MapQTL v6.0 in this study and BSA‐seq in the previous report (Wu et al., 2021b), were selected for further narrowing down experiments.

The QTLs with the *G’ values* larger than 10 were selected for interval narrowing down. To narrow down the QTL regions, five, six, five, and six GenoBaits markers were designed within the intervals of QTLs, F03.2, F03.4, H16.1, and H16.2, respectively. In total, 409 F1 hybrid lines from ‘Zisai Pearl’ × ‘Red Fuji’ were genotyped, and interval mapping was performed using JoinMap 4.0 software (van Ooijen, 2006).

### Candidate gene prediction within QTL regions

Genes within the narrowed‐down QTL regions were screened according to the GDDH13.1 apple genome database, the genes exhibiting genetic variances at the CDS or the 2.0 kb upstream regions between parental cultivars, ‘Zisai Pearl’ and ‘Red Fuji’, were selected as candidates. Then the genes with very low expression levels were culled based on the previous RNA‐seq data (Wu et al., 2021b). Finally, the genes whose functions were previously reported to be closely associated with fruit texture and storability were chosen as key candidate genes.

### DNA/RNA extraction and quantitative real‐time PCR

DNA extraction, total RNA isolation and cDNA synthesis were performed as previously described (Hu et al., 2020). The expression levels of *MdNAC83*, *MdBPM2*, *MdRGLG3*, *MdACO1*, *MdXTH28*, and *MdMANA3* were quantified by RT‐qPCR. The RT‐qPCR analysis was performed using the ABI PRISM 7500 Real‐Time PCR System (Applied Biosystems). The thermal cycling was performed as follows: pre‐denaturation, 30 s at 95°C, 40 cycles of 95°C for 10 s, 60°C for 30 s. The apple *Actin* was used as an internal control; analysis was performed using three technical replicates. The primers used for RT‐qPCR are listed in Table S10.

### GFP/GUS double‐label transient expression assays in tobacco leaf

To construct a GFP/GUS double‐labelled transient expression vector for the *MdNAC83* promoter, the mutated fragments (containing SNP T/C), the 216‐bp deletion fragments and the full‐length promoter fragments of *MdNAC83* were isolated from “Zisai Pearl” and “Red Fuji”, respectively. The segmented construction vector of *MdNAC83* was generated using RT‐qPCR. Promoter fragments were then cloned into the pCambia1301 vector with *Bam*HI restriction sites. The pCambia1301 recombinant plasmid was also co‐transformed into tobacco (*N. benthamiana*) leaves as a positive control, with the reference plasmid PRI101‐GFP to calculate the relative expression. GUS staining was performed using the β‐galactosidase Reporter Gene Staining Kit (Solarbio, China) in three biological replicates and four technical replicates. All primer sequences for vector construction are listed in Table S10.

### Transient over‐expression and VIGS

A 614‐bp *MdNAC83* CDS fragment, a 639‐bp *MdBPM2* CDS fragment, and a 621‐bp *MdRGLG3* CDS fragment were cloned from ‘Golden Delicious’ and ‘Red Fuji’ into the *Eco*RI site of the pTRV2 virus vector as previously described (Li et al., 2017). *Agrobacterium tumefaciens* cells harboring the resultant plasmids were suspended in infiltration buffer supplemented with 150 mM acetosyringone. The inoculum preparations were adjusted to OD_600_ = 1.0. A mixture of *A. tumefaciens* cells harboring pTRV1 and pTRV2 derivatives (1:1 ratio) was infiltrated into “Golden Delicious” apple fruit (Li et al., 2016). The full‐length CDS cDNA sequences (768 bp of *MdNAC83*, 1,242 bp of *MdBPM2*, and 1,107 bp of *MdRGLG3*) were amplified and cloned into the *Bam*HI sites of the pRI101 vector, respectively. The constructs were also infiltrated into ‘Golden Delicious’ and ‘Red Fuji’, respectively. Seven days after infiltration, the phenotype data and samples for the gene expression assay were collected. The assays were performed with at least nine apples for each vector, and the experiments were designed with at least three replicates. The primer pairs are listed in Table S10.

### Apple callus transformation

To determine the impact of the allelic variations in the upstream regions of *MdNAC83* on the promoter activity, the complete *MdNAC83* CDS cDNA sequences were amplified and cloned into the *Bam*HI sites of the PRI101 and RNAi vector, respectively. To construct the plasmid of 35S::*MdBPM2*‐OX, 35S::*MdRGLG3*‐OX, 35S::*MdBPM2‐RNAi*, and 35S::*MdRGLG3‐RNAi*, the full‐length sequence and the specific 200–300‐bp sequence (for anti‐sense) of *MdBPM2* and *MdRGLG3* were inserted into pRI101‐MYC and RNAi vector, respectively. The full‐length sequences of *MdNAC83* were also linked with the *Bam*HI sites of super1300‐GFP vector to construct 35S::*MdNAC83‐GFP* using a one‐step seamless cloning kit (Aidlab Biotechnologies company). The constructs were transformed into apple calli by a previously described method (Jia et al., 2018). The primers used are shown in Table S10.

### Y1H, EMSA, and LUC assay

A Y1H assay was performed according to the protocol of the Matchmaker Gold Yeast One Hybrid System (TaKaRa). The promoter fragments of *MdACO1*, *MdXTH28*, and *MdMANA3* were inserted into the pLaczi vector using the restriction enzyme site *Sal*I. *MdNAC83* was ligated into the pJG4‐5 vector using the restriction enzyme site *Eco*RI, respectively. All primers used are listed in Table S10.

The coding sequence of *MdNAC83* was cloned into the pET‐32a vector that contained a His tag. The recombinant plasmid was transferred into the *Escherichia coli* strain BM Rosetta (DE3) to obtain an *MdNAC83*‐HIS fusion protein. Then, the *MdNAC83* protein was purified using a HIS‐Tagged Protein Purification Kit (TaKaRa). The EMSA reaction was performed using the LightShift Chemiluminescent EMSA kit (ThermoFisher, Scientific), according to methods previously described (Fan et al., 2018).

For checking the binding activity of *MdNAC83* to the promoters of *MdACO1*, *MdXTH28*, and *MdMANA3*, the coding sequence of *MdNAC83* was cloned into the pGreenII 62‐SK vector, acting as an effector vector, and promoters of *MdACO1*, *MdXTH28*, and *MdMANA3* genes were cloned into the pGreenII 0800‐LUC vector, acting as a reporter vector. Then, the vectors were injected into the *N. benthamiana* leaves as described previously (Hellens et al., 2005). The activities of LUC and REN luciferase were measured using the Dual‐Luciferase Assay Kit (Promega) according to the instruction manual. At least six biological replicates were assayed for each combination.

### Y2H, pull‐down, and BiFC assays

The Y2H assays were used for *in vitro* interactions among MdBPM2, MdRGLG3, and MdNAC83. Briefly, the full‐length coding sequences of MdNAC83^A/G^ were inserted into the yeast vectors pGADT7 and pGBKT7. The recombinant plasmids of pGADT7‐MdBPM2^A/T^ and pGBKT7‐MdNAC83^A/G^, pGADT7‐MdRGLG3^G/C^, and pGBKT7‐MdNAC83^A/G^ were co‐transformed into yeast “Y2H Gold.” The yeast was grown on selection medium lacking Trp, Leu, His and Ade (SD/−Trp−Leu−His−Ade) as described previously (Wu et al., 2021b).

For pull‐down assays, MdBPM2^A/T^‐HIS, MdRGLG3^G/C^‐HIS, MdNAC83^A/G^‐GST, and empty GST proteins were used to detect the interaction between MdBPM2^A/T^ and MdNAC83^A/G^. A Pierce HIS Spin Purification Kit was used for pull‐down analysis. The eluted samples were detected by western blotting with GST and HIS antibodies.

For BiFC assays, the coding sequences of MdBPM2^A/T^ and MdNAC83^A/G^ genes were cloned into the CaMV35S::pSPYNE‐nYFP and CaMV35S::pSPYCE‐cYFP vectors, respectively. In addition, recombinant plasmids were injected into the epidermal cells of *N. benthamiana* leaves using an *Agrobacterium*‐mediated method. Yellow fluorescent protein (YFP) signals were detected using a laser confocal microscope (Zeiss LSM 510 META, Jena, Germany).

### Cell‐free and ubiquitination assays

The WT and transgenic apple calli were ground in protein extraction buffer containing 25 mM Tris (pH 7.4), 0.5 mM EDTA, 10 mM NaCl, 10 mM MgCl_2_, 5 mM DTT, and 4 mM phenylmethanesulfonyl fluoride (PMSF) and co‐incubated with fusion MdNAC83‐GST protein at 22°C. Samples were collected at 0, 30, 60, and 90 min. For the proteasome inhibitor experiments, three types of apple calli were treated with 50 µM MG132 and then extracted. Subsequently, the extracts were co‐incubated with MdNAC83‐GST protein. The results were assessed by western blotting with anti‐GST and anti‐actin antibodies (Abcam, Shanghai, China).

For the *in vitro* ubiquitination assay, the MdBPM2, MdRGLG3 and MdNAC83 coding sequences were cloned into the pGEX‐32a or pGEX‐4T‐1 vector and expressed in *Escherichia coli* (BL21) cells to produce fusion proteins with a His tag (MdBPM2‐His, MdRGLG3‐His) or GST tag (MdNAC83‐GST). Recombinant UBE1 (E1; 0.75 mg; UBBiotech), human E2 (E2; 6 mg; UBBiotech), MdBPM2‐HIS and MdRGLG3‐HIS (E3), ubiquitin (50 mg; UBBiotech), and purified MdNAC83‐GST were mixed in the reaction buffer (1 mM ATP, 60 mM DTT, 500 mM Tris, and 100 mM MgCl_2_). The reactions were completed at 37°C for 3 h. Ubiquitinated MdBPM2 and MdRGLG3 were detected in a western blot with the anti‐His monoclonal (CWBio) and anti‐ubiquitin (UBBiotech) antibodies.

For ubiquitination assay *in vivo*, Super1300::MdNAC83‐GFP/WT and Super1300::MdNAC83‐GFP/35S::MdBPM2‐MYC transgenic apple calli were extracted, and a Pierce classic IP kit (Thermo) was used to immunoprecipitate 35S::MdBPM2‐MYC with an anti‐MYC antibody, and the immunoprecipitated protein was analyzed by immunoblot with anti‐Ubi and anti‐MYC antibodies.

### Marker development and genotyping using the GenoBaits assay

SNP and InDel markers were developed within each QTL region identified via both the map‐based QTL strategy in this study and the previous BSA‐seq for FF, FC, FFR, and FCR (Wu et al., 2021b). Finally, in total, 257 GenoBaits markers were developed, including 56 markers designed based on the SNPs and InDels we previously reported (Wu et al., 2021b). Sequence‐specific GenoBaits probes (120 nt) were designed flanking the target variations, then, the GenoBaits probes for different targets were mixed up and modified (Guo et al., 2019). DNA library construction and probe hybridization were performed according to Xu et al. (2020). After Illumina high‐throughput next‐generation sequencing, the sequencing data were processed, and the marker genotypes were obtained following the procedure of Guo et al. (2019).

### Marker genotype effect estimation and GAP modeling

The training population for GAP modeling included 612 *Malus* germplasm accessions and 1,191 hybrid plants from three biparental cross populations. Marker genotyping was performed using the strategy described by the previous authors (Guo et al., 2019; Liu et al., 2022b). Reliable genotype‐phenotype one‐to‐one matched data for FF, FC, FFR and FCR were obtained in 1,382, 1,370, 1,182, and 1,187 individuals of the training population, respectively. Marker genotype effects or joint effects of marker genotype combinations were estimated by the deviation between the average observed phenotype values (OPV) of the hybrid lines with the same genotype and the overall mean phenotype value of the training populations (Zheng et al., 2020b; Wu et al., 2021b; Shen et al., 2022). For an individual in the training population, the genotype predicted value (GPV) of a trait was calculated by the sum of the genotype effects and the joint effects of all markers and the population mean phenotype value (Zheng et al., 2020b). The prediction accuracy of GPV was evaluated by the Pearson's correlation coefficient between GPV and OPV of individuals in the test population (Muranty et al., 2015). Five‐fold cross‐validation of the GAP models was performed as previously described (Zheng et al., 2020b; Wu et al., 2021b; Shen et al., 2022; Wen et al., 2024).

### Genetic composition and structure analysis

All the 257 GenoBaits markers and all the 612 *Malus* accessions were used for genetic structure analysis (http://pophelper.com). Eighteen GenoBaits markers with relatively large effects on FCR were chosen for genetic composition analysis. Two hundred and ninety‐two hybrid lines from ‘Jonathan’ × ‘Golden Delicious’ and all the 612 *Malus* accessions were used for genetic composition analysis, because hybrid plants derived from ‘Zisai Pearl’ were interspecific hybrids and were not included. Genotype frequencies of each marker were calculated and compared between *Malus* species.

### Statistical analysis

The data from each experiment were collected based on three biological replicates and three technical replicates. Statistical analysis was performed using DPS software.

## CONFLICTS OF INTEREST

The authors declare no conflicts of interest.

## AUTHOR CONTRIBUTIONS

B.W. performed the experiments, analyzed the data, and wrote the draft manuscript. X.Z. and Z.H. designed and supervised the experiments. X.Z. revised the manuscript. F.S. performed bioinformatic analysis. Z.Z. and W.R. collected partial phenotype data. Y.W. and T.W. prepared the plant materials. All authors read and approved the manuscript.

## Supporting information

Additional Supporting Information may be found online in the supporting information tab for this article: http://onlinelibrary.wiley.com/doi/10.1111/jipb.70044/suppinfo

**Figure S1.** Narrowing down of the QTL F03.2 interval and peptide folding of *MdNAC83*
**Figure S2.** The effects of genetic variations at the promoter and coding sequence of *MdNAC83* on apple fruit storability
**Figure S3.** The genotype effects of *MdBPM2* SNP657 T/A and *MdRGLG3* SNP167 C/G on apple storability
**Figure S4.** Weighted correlation network analysis (WGCNA) and co‐expression analysis to predict downstream target genes of *MdNAC83*
**Figure S5.** Transiently over‐expression or virus‐induced gene silencing of *MdBPM2* in 'Golden Delicious' and 'Red Fuji'.
**Figure S6.** Fruit ethylene production after 120 days of cold storage using 36 randomly chosen hybrid lines with different genotype combinations
**Figure S7.** Transient over‐expression or virus‐induced gene silencing of *MdRGLG3* in 'Golden Delicious' and 'Red Fuji'.
**Figure S8.** MdRGLG3 self‐ubiquitination activity
**Figure S9.** Linear regression between GPV and OPV representing the prediction accuracy of additive genomics‐assisted prediction models for apple storability
**Figure S10.** Genetic structure analysis for apple fruit storability using 257 SNP/Indel markers in a natural population of 612 apple accessions from six *Malus* species
**Figure S11.** Phylogenetic analysis and FPKM value of *MdBPM2* and *MdRGLG3*

**Table S1.** QTLs for apple flesh firmness and flesh crispness at harvest identified using MapQTL6.0 in a hybrid population of 'Zisai Pearl' × 'Red Fuji'
**Table S2.** Marker genotypes for narrowing down the intervals of QTLs F03.4, H16.1, H16.2 and F03.2 for apple flesh firmness retainability using a hybrid population (*n* = 409) of 'Zisai Pearl' × 'Red Fuji'
**Table S3.** Genes within the narrowed‐down regions of QTLs F03.4, H16.1, and H16.2 for apple flesh firmness retainability
**Table S4.** Genotypes of allelic variations of *MdNAC83, MdBPM2, MdRGLG3, MdACO1, MdMANA3*, and *MdXTH28* in the parental cultivars, 'Golden Delicious', 'Red Fuji', and 'Zisai Pearl'
**Table S5.** Estimates of marker genotype effects on apple FF, FC, FFR and FFC in the training population
**Table S6.** List of the 146 apple fruit storability‐associated genes identified by RNA‐seq analysis and QTL analysis
**Table S7.** Joint effects of genotype combinations of interacting functional markers on apple FF, FC, FFR, and FCR, respectively
**Table S8.** Genetic composition analysis of 18 markers with large genotype effect on FCR in six *Malus* species
**Table S9.** Joint effects of genotype combinations of variations in *MdBPM2/MdNAC83/MdRGLG3* and *MdERF3/MdERF118* on apple fruit storability traits
**Table S10.** The primer sequences used for qRT‐PCR, molecular interaction, and gene cloning

## Data Availability

Sequence data were based on online databases (https://www.uniprot.org/ and https://www.rosaceae.org/species/malus/malus × domestica, GDR). All BSA‐seq raw data have been deposited in the NCBI Sequence Read Archive (SRA) under the accession number PRJNA650592. The accession numbers for the genes in this study are as follows: *MdNAC83* (MD16G1125800), *MdBPM2* (MD03G1269700), *MdRGLG3* (MD16G1282700), *MdACO1* (MD10G1328100), *MdXTH28* (MD16G1014000) and *MdMANA3* (MD02G1129000).
