# Supplementary material for: Natural variations in MdBPM2/MdRGLG3‐MdNAC83 network controlling the quantitative segregation of apple fruit storability
Source: J Integr Plant Biol. 2025 Oct 1;68(1):169–90. doi: 10.1111/jipb.70044 (PMC12782892; doi:10.1111/jipb.70044)
Supplement: Supplementary file 1 — Figure S1. Narrowing down of the QTL F03.2 interval and peptide folding of MdNAC83 Figure S2. The effects of genetic variations at the promoter and coding sequence of MdNAC83 on apple fruit storability Figure S3. The genotype effects of MdBPM2 SNP657 T/A and MdRGLG3 SNP167 C/G on apple storability Figure S4. Weighted correlation network analysis (WGCNA) and co‐expression analysis to predict downstream target genes of MdNAC83 Figure S5. Transiently over‐expression or virus‐induced gene silencing of MdBPM2 in 'Golden Delicious' and 'Red Fuji'. Figure S6. Fruit ethylene production after 120 days of cold storage using 36 randomly chosen hybrid lines with different genotype combinations Figure S7. Transient over‐expression or virus‐induced gene silencing of MdRGLG3 in 'Golden Delicious' and 'Red Fuji'. Figure S8. MdRGLG3 self‐ubiquitination activity Figure S9. Linear regression between GPV and OPV representing the prediction accuracy of additive genomics‐assisted prediction models for apple storability Figure S10. Genetic structure analysis for apple fruit storability using 257 SNP/Indel markers in a natural population of 612 apple accessions from six Malus species Figure S11. Phylogenetic analysis and FPKM value of MdBPM2 and MdRGLG3 [file JIPB-68-169-s001.docx]

**Natural variations in MdBPM2/MdRGLG3-MdNAC83 network controlling the quantitative segregation of apple fruit storability**

Bei Wu, Fei Shen, Ziying Zhou, Wenhui Ren, Yi Wang, Ting Wu, Zhenhai Han, Xinzhong Zhang^*^

College of Horticulture, China Agricultural University, Beijing 100193, China

^*^Correspondence: Xinzhong Zhang ([zhangxinzhong999@126.com](mailto:zhangxinzhong999@126.com)).

**Supporting information**


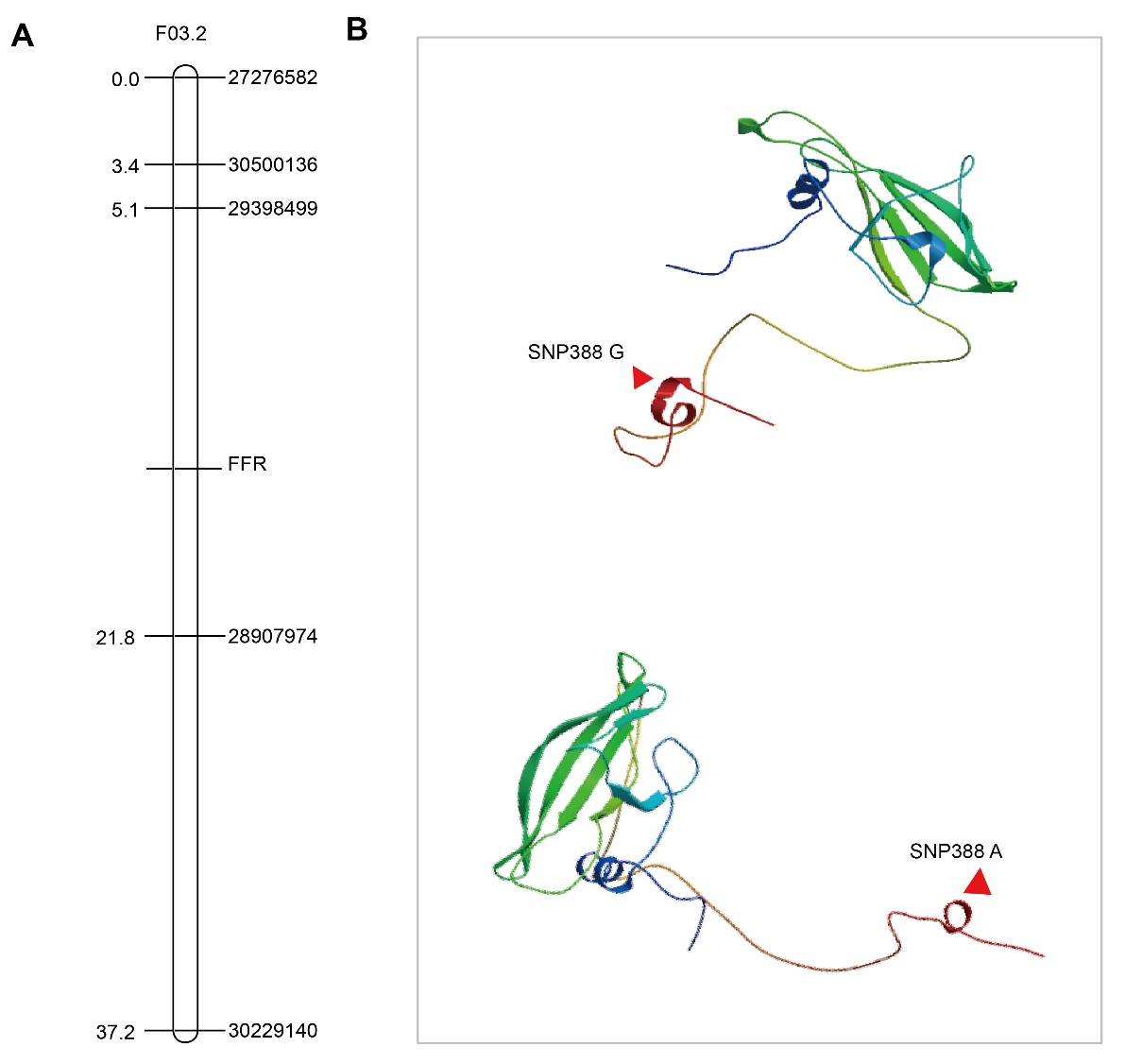


**Figure S1. Narrowing down of QTL F03.2 interval and peptide folding of *MdNAC83.* (A)** QTL F03.2 interval was narrowed down by using a biparental cross population (*Malus asiatica* Nakai ‘Zisai Pearl’ × *M. domestica* Borkh. ‘Red Fuji’). **(B)** Peptide folding of *MdNAC83* was altered by SNP388 A marked with a filled red triangle. FFR, flesh firmness retainability.

**
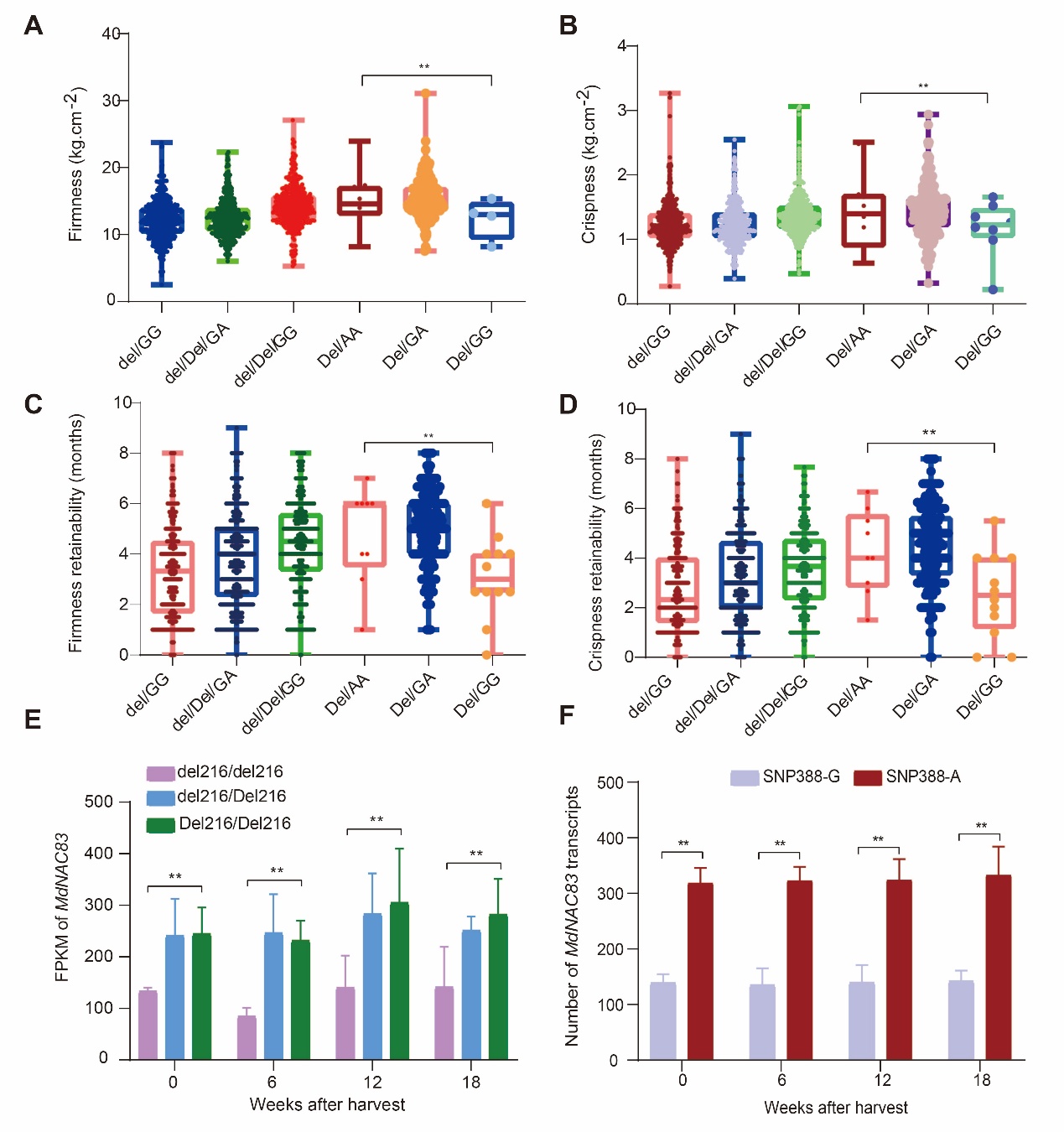
**

**Figure S2. The effects of genetic variations at promoter and coding sequence of *MdNAC83* on apple fruit storability. (A-D)** The joint effects of genotype combinations of *MdNAC83* Del216 and SNP388 G/A on apple fruit flesh firmness at harvest **(A)**, flesh crispness at harvest **(B)**, flesh firmness retainability **(C)**, and flesh crispness retainability **(D)**, respectively. **(E)** Previous RNA-seq data showing the expression of *MdNAC83* in apple mesocarp of hybrid lines with different Del216 genotypes during postharvest cold storage. Three hybrid lines for each Del216 genotype were used. **(F)** Previous RNA-seq data showing the abundance of transcripts with different alleles of *MdNAC83* SNP388 G/A in the mesocarp of three hybrid lines with *MdNAC83* Del216:del216 heterozygous genotype.

**
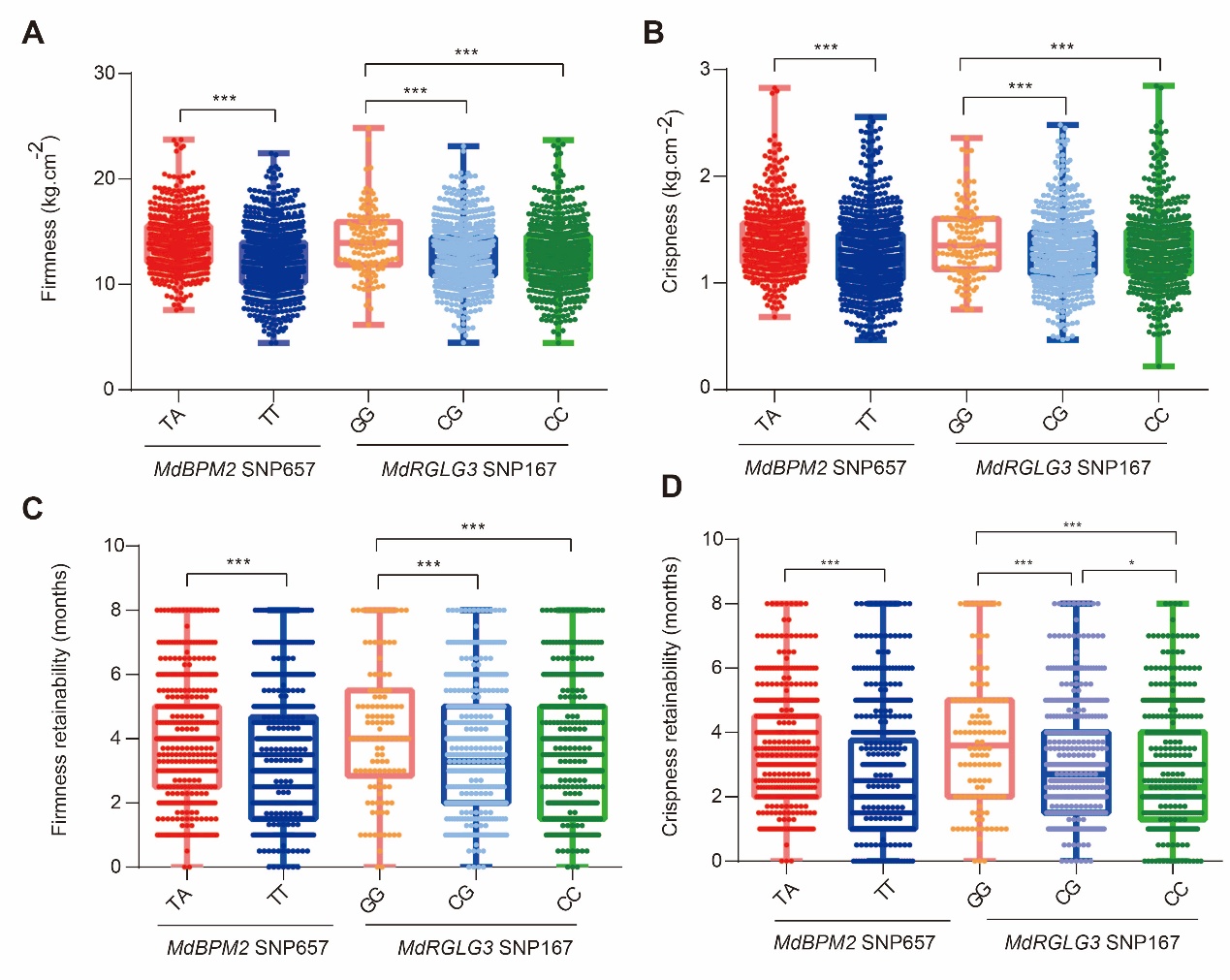
**

**Figure S3. The genotype effects of *MdBPM2* SNP657 T/A and *MdRGLG3* SNP167 C/G on apple storability. (A-D)** The genotype effects of *MdBPM2* and *MdRGLG3* on flesh firmness at harvest **(A)**, flesh crispness at harvest **(B)**, flesh firmness retainability **(C)**, and flesh crispness retainability **(D)**, respectively.

**
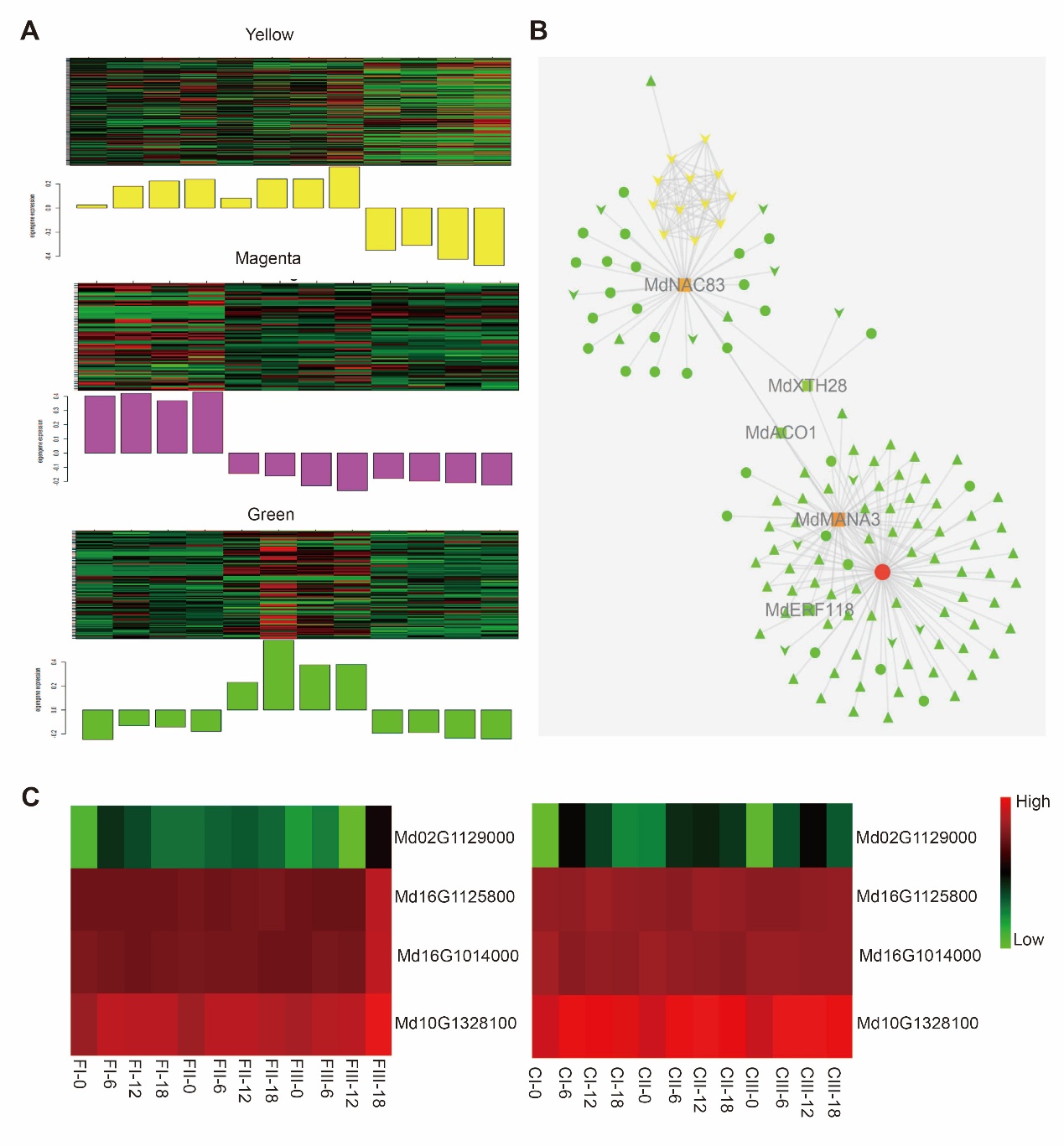
**

**Figure S4. Weighted correlation network analysis (WGCNA) and co-expression analysis to predict downstream target genes of *MdNAC83*. (A)** WGCNA modules significant differential gene expressions between fruit samples with different storability. **(B)** Co-expression network showing genes co-expressed with *MdNAC83*. **(C)** Heatmaps show the expression patterns of *MdNAC83* and the candidate target genes during cold storage. FIII/CIII, FII/CII and FI/CI represent ﬂesh ﬁrmness (F) and crispness (C) retainability phenotype type III, II and I, respectively. Samples were collected at 0, 6, 12, and 18 weeks of cold storage (Wu et al., 2021b).

**
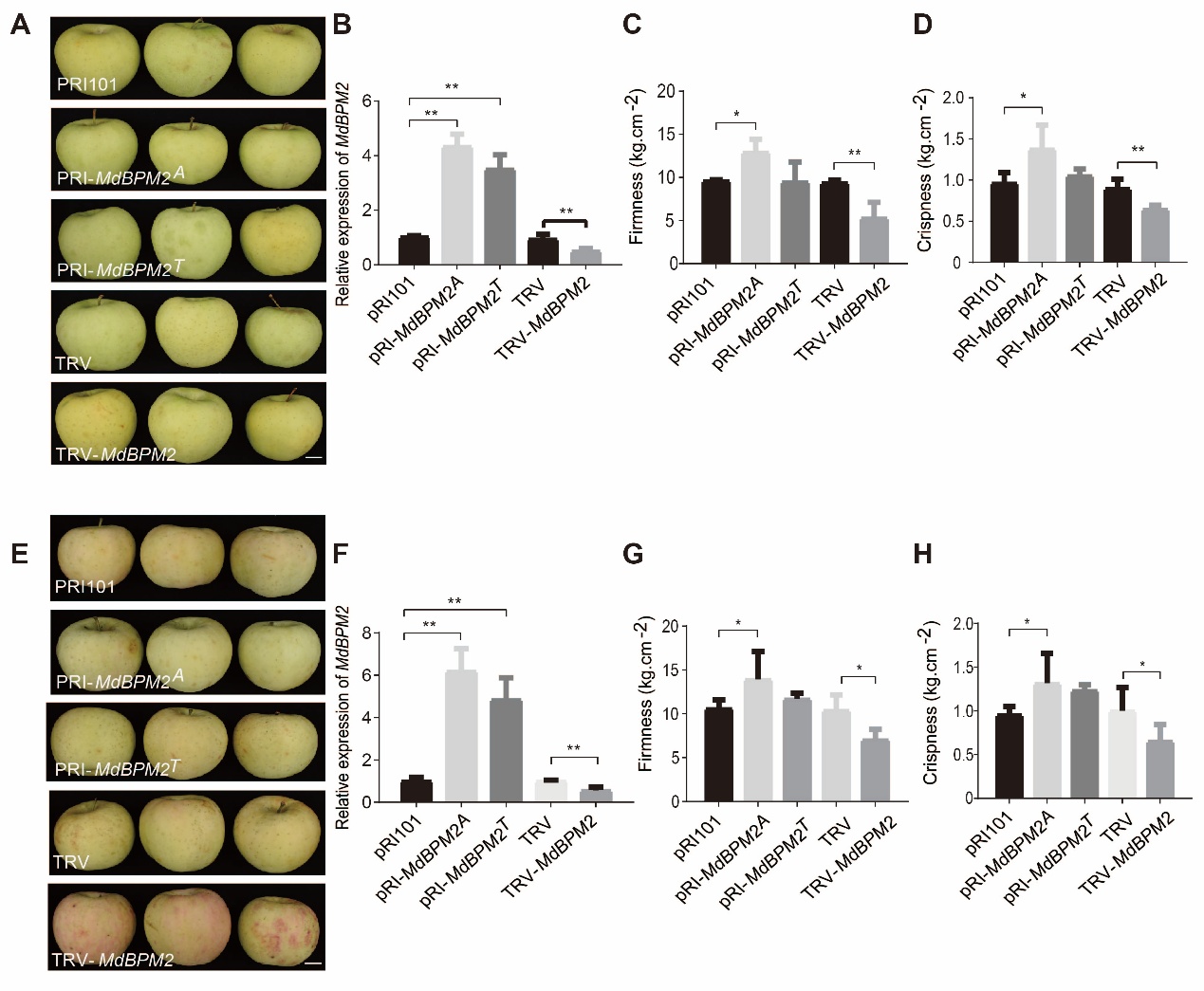
**

**Figure S5. Transiently over-expressing or virus-induced gene silencing of *MdBPM2* in ‘Golden Delicious’ and ‘Red Fuji’.** **(A, E)** Photographs showing changes in appearance phenotype after transient transformations in ‘Golden Delicious’ and ‘Red Fuji’, respectively. Scale bars, 3mm. **(B, F)** The expression of *MdBPM2* over-expressing (*pRI-MdMdBPM2*) or silencing (*TRV-MdBPM2*) in ‘Golden Delicious’ and ‘Red Fuji’, respectively. **(C, D, G, H)** Changes of flesh firmness **(C, G)** and flesh crispness **(D, H)** in transiently transformed apples with empty vector ( pRI101 and TRV), *pRI-MdBPM2,* and *TRV-MdBPM2.* Asterisks indicate statistical signiﬁcance (**P < 0.05, **P < 0.01, ***P < 0.001*).

**
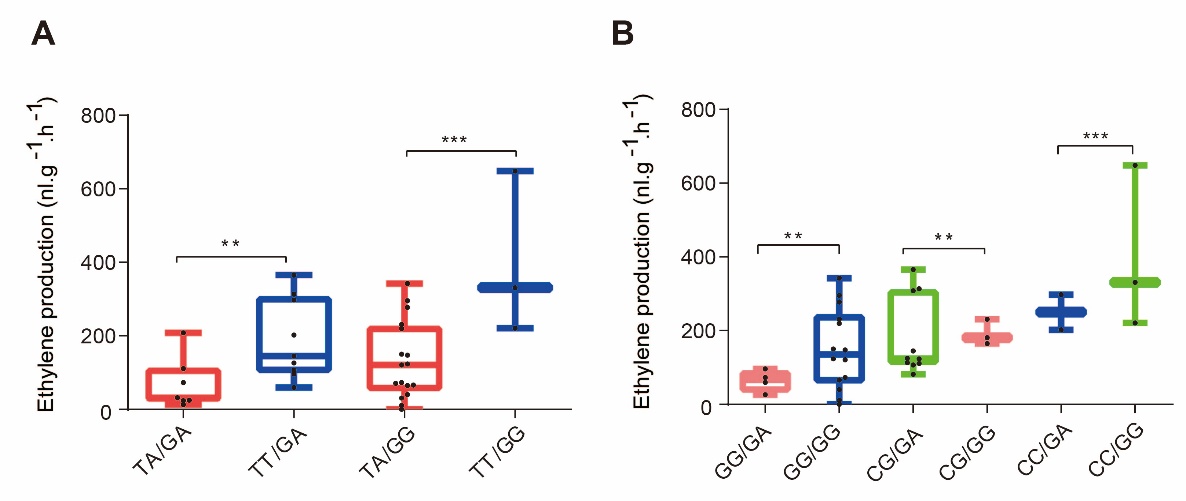
**

**Figure S6. Fruit ethylene production after 120 days of cold storage by using 36 randomly chosen hybrid lines with different genotype combinations. (A)** *MdNAC83* SNP388 G/A and *MdBPM2* SNP657 T/A. **(B)** *MdNAC83* SNP388 G/A and *MdRGLG3* SNP167 C/G

**
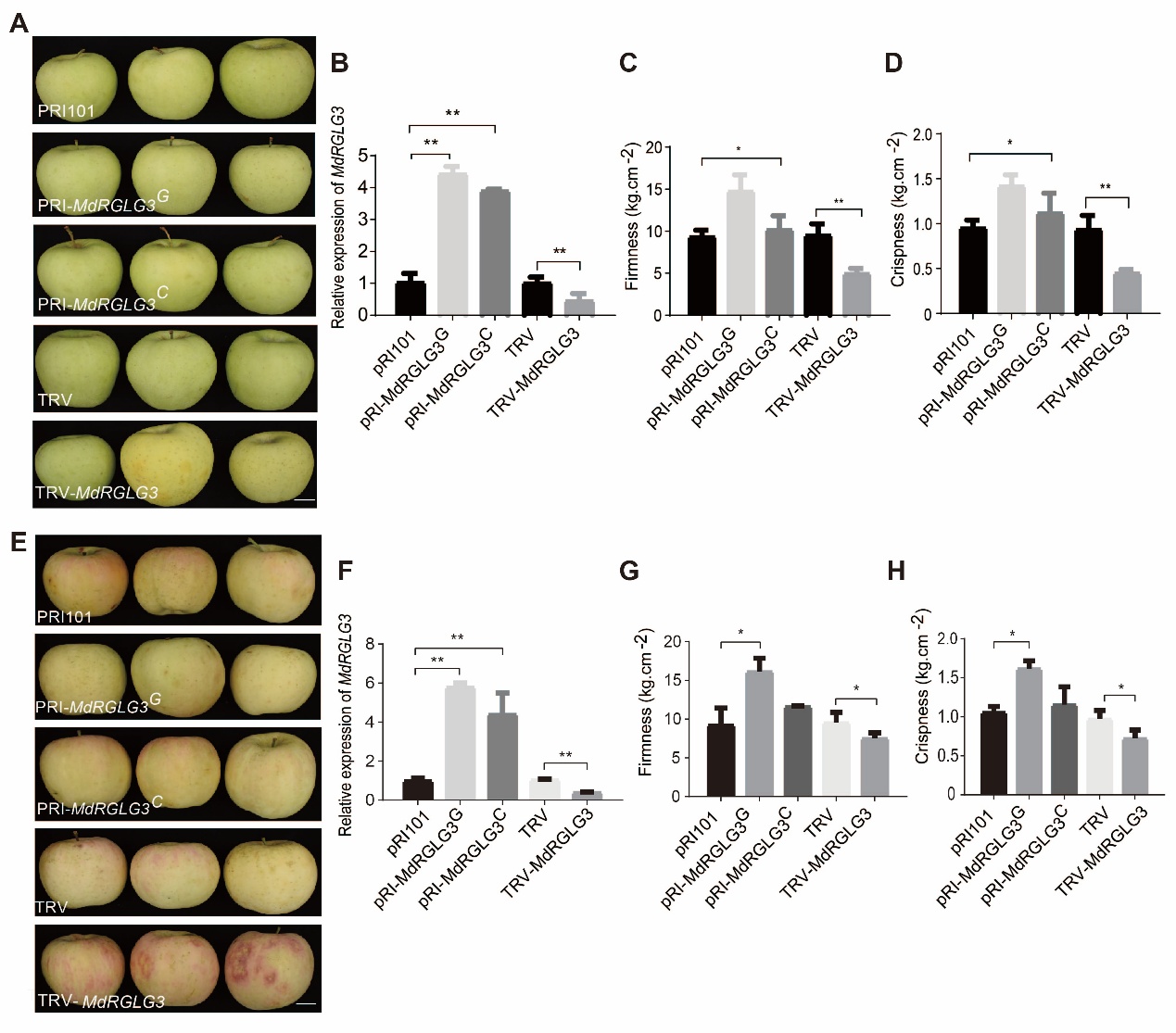
**

**Figure S7. Transiently over-expressing or virus-induced gene silencing of *MdRGLG3* in ‘Golden Delicious’ and ‘Red Fuji’. (A, E)** Photographs showing changes in appearance phenotype after transient transformations in ‘Golden Delicious’ and ‘Red Fuji’, respectively. Scale bars, 3mm. **(B, F)** The expression of *MdRGLG3* over-expressing (*pRI-MdMdRGLG3*) or silencing (*TRV-MdRGLG3*) in ‘Golden Delicious’ and ‘Red Fuji’, respectively. **(C, D, G, H)** Changes in flesh firmness **(C, G)** and flesh crispness **(D, H)** in transiently transformed apples with empty vector (pRI101 and TRV), *pRI-MdRGLG3,* and *TRV-MdRGLG3*. Asterisks indicate statistical signiﬁcance (**P < 0.05, **P < 0.01, ***P < 0.001*).

**
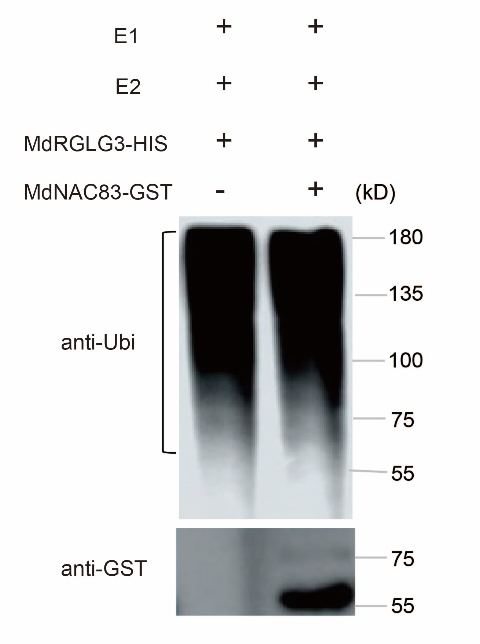
**

**Figure S8. MdRGLG3 self-ubiquitination activity**. Anti-GST and anti-Ubi antibodies were used to examine immunoprecipitation.

**
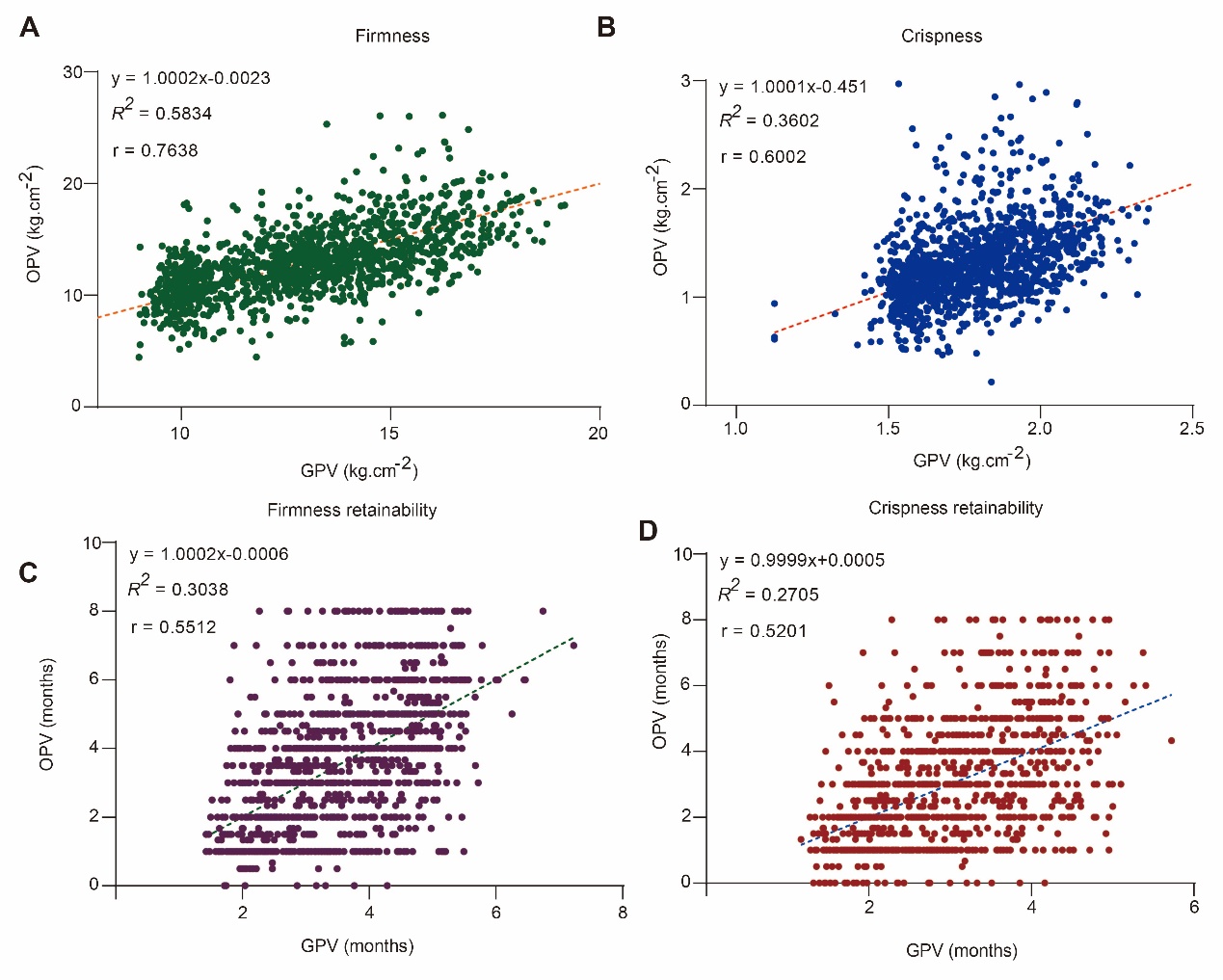
**

**Figure S9. Linear regression between GPV and OPV representing the prediction accuracy of additive genomics-assisted prediction models for apple storability.** **(A-D)** The prediction accuracy of additive GAP models for flesh firmness at harvest **(A)**, flesh crispness at harvest **(B)**, flesh firmness retainability **(C)**, and flesh crispness retainability **(D)** by addition of functional markers to the marker panel.

**
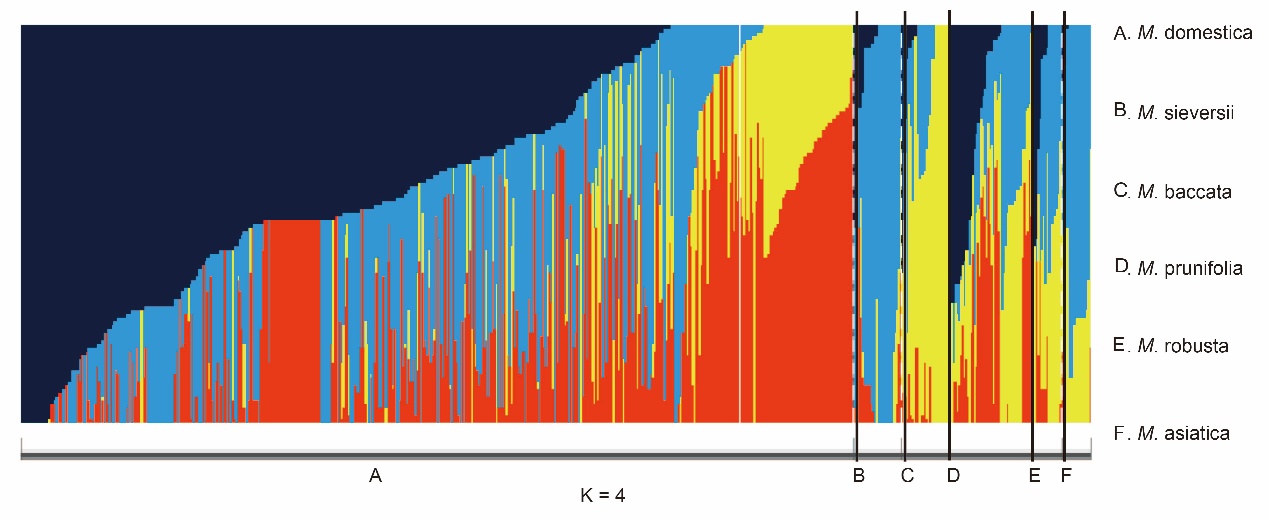
**

**Figure S10. Genetic structure analysis for apple fruit storability by using 257 SNP/Indel markers in a natural population of 612 apple accessions from 6 *Malus* species.**

**
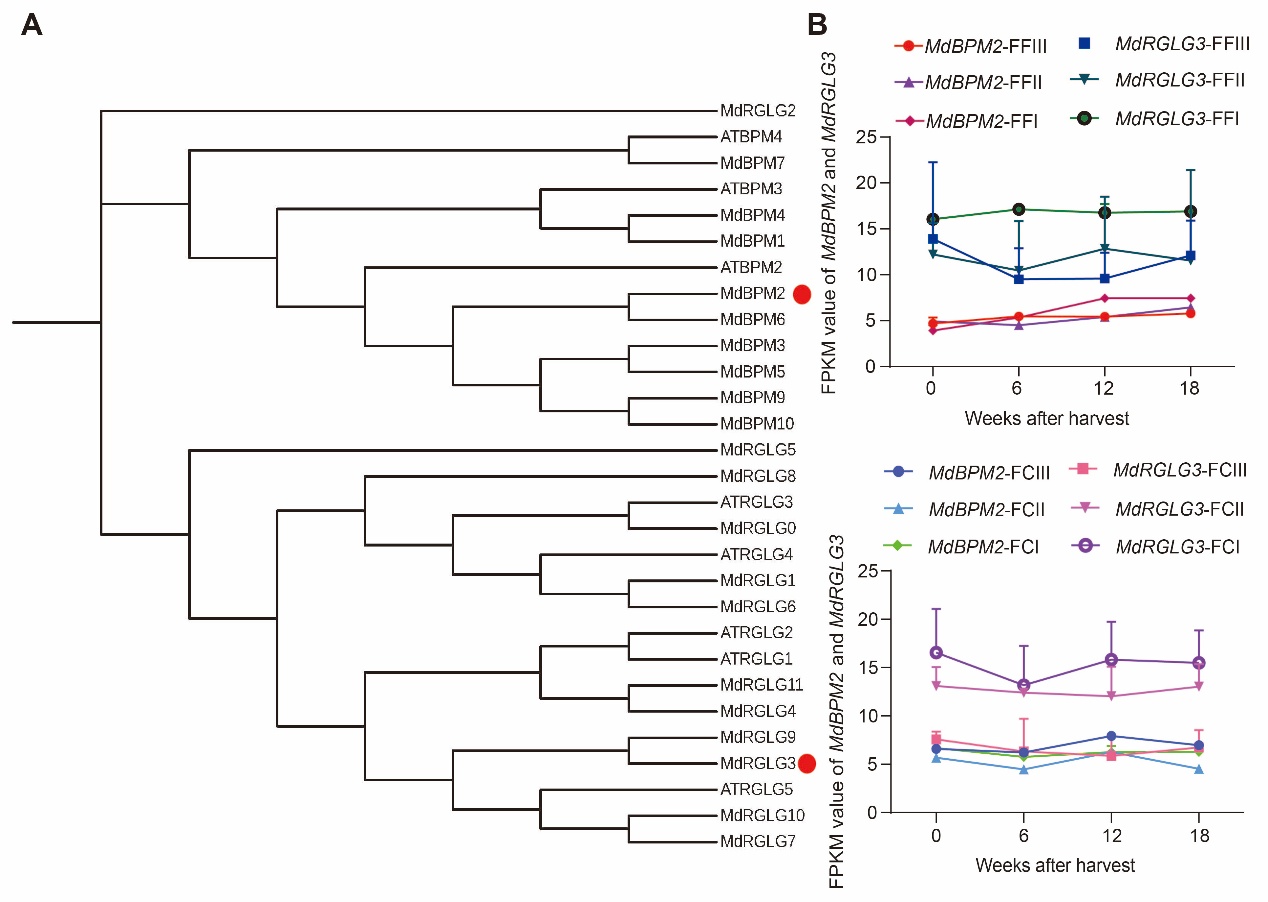
**

**Figure S11. Phylogenetic analysis and FPKM value of *MdBPM2* and *MdRGLG3.*** **(A)** The phylogenetic analysis of *MdBPM* and *MdRGLG* super families. **(B)** FPKM value of *MdBPM2* and *MdRGLG3*.
